# Mitochondrial genomes of blister beetles (Coleoptera, Meloidae) and two large intergenic spacers in *Hycleus* genera

**DOI:** 10.1186/s12864-017-4102-y

**Published:** 2017-09-06

**Authors:** Chao Du, Lifang Zhang, Ting Lu, Jingnan Ma, Chenjuan Zeng, Bisong Yue, Xiuyue Zhang

**Affiliations:** 1 Key Laboratory of Bio-resources and Eco-environment (Ministry of Education), College of Life Sciences, Sichuan University, Chengdu, 610064 People’s Republic of China; 2 Sichuan Key Laboratory of Conservation Biology on Endangered Wildlife, College of Life Sciences, Sichuan University, Chengdu, 610064 People’s Republic of China; 3 Sichuan Key Laboratory of Medicinal American Cockroach, Chengdu, 610041 People’s Republic of China; 4 Nanchong Vocational and Technical College, Nanchong, 637131 Sichuan People’s Republic of China

**Keywords:** Blister beetle, Meloidae, Mitochondrial genomes, Large intergenic spacer, Evolutionary mechanism, Molecular phylogeny

## Abstract

**Background:**

Insect mitochondrial genomes (mitogenomes) exhibit high diversity in some lineages. The gene rearrangement and large intergenic spacer (IGS) have been reported in several Coleopteran species, although very little is known about mitogenomes of Meloidae.

**Results:**

We determined complete or nearly complete mitogenomes of seven meloid species. The circular genomes encode 13 protein-coding genes (PCGs), 22 transfer RNAs (tRNAs) and two ribosomal RNAs (rRNAs), and contain a control region, with gene arrangement identical to the ancestral type for insects. The evolutionary rates of all PCGs indicate that their evolution is based on purifying selection. The comparison of tRNA secondary structures indicates diverse substitution patterns in Meloidae. Remarkably, all mitogenomes of the three studied *Hycleus* species contain two large intergenic spacers (IGSs). IGS1 is located between *trnW* and *trnC*, including a 9 bp consensus motif. IGS2 is located between *trnS2 (UCN)* and *nad1*, containing discontinuous repeats of a pentanucleotide motif and two 18-bp repeat units in both ends. To date, IGS2 is found only in genera *Hycleus* across all published Coleopteran mitogenomes. The duplication/random loss model and slipped-strand mispairing are proposed as evolutionary mechanisms for the two IGSs (IGS1, IGS2). The phylogenetic analyses using MrBayes, RAxML, and PhyloBayes methods based on nucleotide and amino acid datasets of 13 PCGs from all published mitogenomes of Tenebrionoids, consistently recover the monophylies of Meloidae and Tenebrionidae. Within Meloidae, the genus *Lytta* clusters with *Epicauta* rather than with *Mylabris*. Although data collected thus far could not resolve the phylogenetic relationships within Meloidae, this study will assist in future mapping of the Meloidae phylogeny.

**Conclusions:**

This study presents mitogenomes of seven meloid beetles. New mitogenomes retain the genomic architecture of the Coleopteran ancestor, but contain two IGSs in the three studied *Hycleus* species. Comparative analyses of two IGSs suggest that their evolutionary mechanisms are duplication/random loss model and slipped-strand mispairing.

**Electronic supplementary material:**

The online version of this article (10.1186/s12864-017-4102-y) contains supplementary material, which is available to authorized users.

## Background

The animal mitochondrial genome (mitogenome) is an informative model for phylogenetics, molecular evolution, and comparative genomic research, due to its simple genetic structure, maternal inheritance, and high evolutionary rate properties [1–3]. The mitogenome has been increasingly used to analyze the phylogeny and evolution of the highly diverse and rapidly radiating insects [4–8]. The typical mitogenome of metazoans is a circular molecule encoding a conserved set of 37 genes for 13 protein-coding genes (PCGs), two ribosomal RNA (rRNA) genes, and 22 transfer RNA (tRNA) genes, and comprising a non-coding control region [3]. The mitogenome commonly displays exceptional economy of organization evidenced by lacking introns, few intergenic spacers, incomplete stop codons, and even overlapping genes [9]. However, the extremely diverse Insecta also exhibits high diversity in their mitochondrial genomes, such as gene rearrangements and/or long non-coding regions (except the control region) in some lineages within Hymenoptera [10–14], Hemiptera [15–17], Dictyoptera [18, 19], Diptera [20], Orthoptera [21], Thysanoptera [22], Psocoptera [23], and Phthiraptera [24]. Within the order Coleoptera [25], most beetles retain the same gene content and gene organization as the hypothesized ancestral mitogenome for Insecta [26], while a few possess gene rearrangements of tRNAs [7, 27, 28] and PCGs [29] in their mitogenomes. For example, the mitogenome of the firefly, *Pyrocoelia rufa* contains a 1724-bp large intergenic spacer (IGS) composed of tandem repeat units [5]. Non-coding intergenic spacers were also found in the mitogenomes of other beetles (*Trachypachus holmbergi*, *Priasilpha obscura*, and *Cyphon sp.*) [30]. Although a recent study reported that *Hycleus chodschenticus* has a large intergenic spacer [31], very little is known about the rearrangement and large non-coding regions in Meloidae mitogenomes.

Meloidae is a medium sized family within Coleoptera Tenebrionoidea, containing more than 3000 species within approximately 125 genera [32]. Meloids are commonly referred to as blister beetles due to a defensive secretion, cantharidin. Cantharidin is an intoxicant that can be used for the removal of warts and may be effective in the treatment of primary liver cancer, leucocytopenia, chronic liver disease, neurodermatitis and other major illnesses [33, 34]. The medicinal properties of cantharidin, hypermetamorphosis development, and parasitoid habit of Meloidae species have been extensively researched [35, 36]. However, the mitogenomes of Meloidae species is less well researched. Two of the few studies of Meloidae mitogenomes found that *Epicauta chinensis* and *Lytta caraganae* retained the ancestral model of the insect mitogenome, without any gene rearrangement or long non-coding regions [31, 37]. Of the approximately 3000 species within Meloidae, only three complete mitogenomes (of *Epicauta chinensis*, *Lytta caraganae*, and *Hycleus chodschenticus*) have been described (excluding another without any description; *Mylabris sp.*, JX412732.1). The lack of research considerably limits the genomic comparisons and molecular phylogenetic studies of Meloidae. Thus we believed there was an urgent need to explore the mitogenome evolution in the diverse family of Meloidae.

Consequently, we determined the mitogenomes of seven blister beetle species, representing four meloid genera. These species were *E. gorhami*, *E. tibialis*, *L. caraganae*, *Mylabris aulica*, *H. phaleratus*, *H.marcipoli*, and *H. cichorii*. We described the general features of the newly sequenced mitogenomes from the seven species and analyzed two IGSs in all *Hycleus* species to explore their evolutionary mechanisms. In addition, we attempted to assess the possibility of the IGS2 to be a molecular marker and indicators of phylogenetic relationships within Meloidea, based on mitogenomic datasets. The mitogenomes of the seven meloids will significantly add to the knowledge of Meloidae taxonomy, phylogeny, and evolution.

## Results

### Genome content and gene organization

**Fig. 1:**
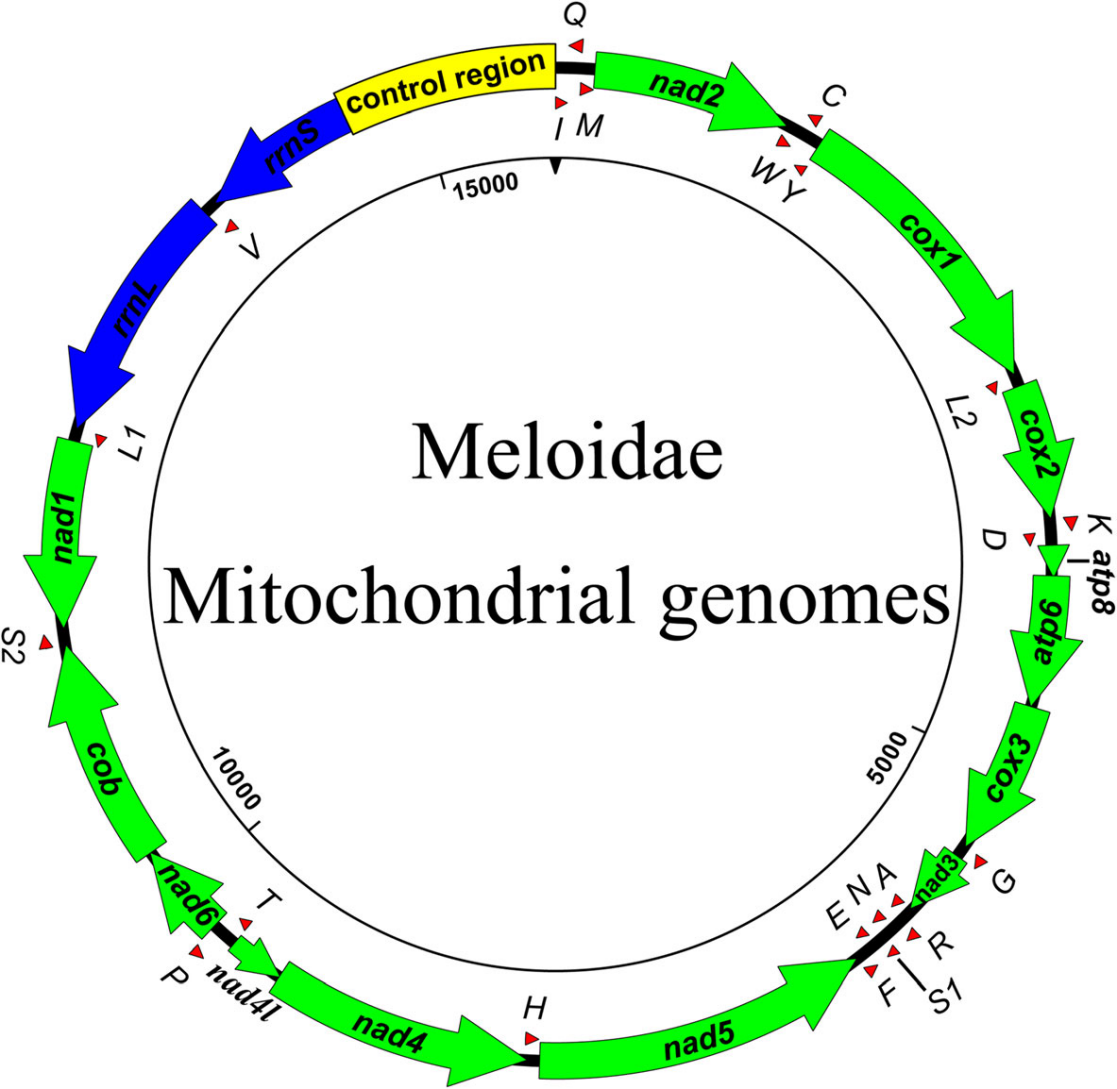
Circular map of the meloid mitochondrial genomes. tRNA genes are abbreviated to the capital letter of their coding amino acid, *L*, *L2*, *S* and *S2* indicate *trnL1 (CUN)*, *trnL2 (UUR)*, *trnS1 (AGN)*, and *trnS2 (UCN)*. Arrows indicate the direction of transcription

This study presents six complete mitogenomes and one nearly complete mitogenome (*H. cichorii*) with the absence of the control region and three tRNAs (*trnI*, *trnQ* and *trnM*). The total lengths of complete mitogenomes range from 15,633 to 16,003 bp. All seven new sequences were submitted to GenBank under the accession numbers listed in Table 1. Every mitogenome of the seven meloid species is a circular DNA molecule, encoding the typical 37 genes including 13 PCGs, 22 tRNAs, two rRNAs, and a putative control region. The major strand (J strand) carries most of the genes (9 PCGs and 14 tRNAs), while the remaining genes are encoded on the minor strand (N strand) (Fig. 1).

**Table 1:** Locality information and accession numbers of meloid species employed in this study

Species	Locality	Size (bp)	A + T content	Accession
*Mylabris aulica*	Dongsheng, Inner Mongolia, China	15,758	68.98	KX161860
*Hycleus phaleratus*	Luodian, Guizhou, China	16,003	69.94	KX161858
*Hycleus marcipoli*	Beihai, Guangdong, China	15,923	71.94	KX161857
*Hycleus cichorii*	Luodian, Guizhou, China	14,370	69.13	KX161856
*Epicauta tibialis*	Beihai, Guangdong, China	15,816	67.53	KX161855
*Epicauta gorhami*	Huaying, Shaanxi, China	15,691	69.18	KX161854
*Lytta caraganae*	Dongsheng, Inner Mongolia, China	15,633	71.13	KX161859

All PCGs of the seven mitogenomes use typical ATN start codons. Conventional stop codons TAA/TAG are assigned to most of the PCGs, but the *cox1*, *cox2*, *nad5* and *nad4* genes of all meloids terminate with the incomplete stop codon T. This terminator is adopted by *cox3* genes of *Epicauta* and *Lytta* species (Additional files 1, 2, 3, 4, 5, 6 and 7: Tables S1–S7). The A + T - rich regions of meloid mitogenomes range from 1015 to 1201, with the location between *rrnS* and *trnI* (Table 2). The poly-T stretch (15 bp) was detected in control regions of all meloids, but without tandem repeats.

**Table 2:** Annotations of the seven meloid mitochondrial genomes

Gene	Strand^a^	*Mylabris aulica*	*Hycleus phaleratus*	*Hycleus marcipoli*	*Hycleus cichorii*	*Epicauta tibialis*	*Epicauta gorhami*	*Lytta caraganae*
*trnI*	J	1–66	1–65	1–65		1–66	1–66	1–66
*trnQ*	N	64–132 (−3)	63–131 (−3)	63–131 (−3)		64–132 (−3)	64–132 (−3)	64–132 (−3)
*trnM*	J	132–200 (−1)	131–199 (−1)	131–199 (−1)		132–200 (−1)	132–200 (−1)	132–200 (−1)
*nad2*	J	201–1214	200–1213	200–1213	18–1031	201–1214	201–1214	201–1214
*trnW*	J	1213–1281 (−2)	1216–1281 (2)	1213–1280 (−1)	1034–1099 (2)	1213–1279 (−2)	1213–1280 (−2)	1213–1280 (−2)
*trnC*	N	1284–1347 (2)	1343–1406 (62)	1337–1400 (56)	1139–1202 (39)	1279–1342 (−1)	1280–1343 (−1)	1280–1343 (−1)
*trnY*	N	1350–1413 (−2)	1409–1474 (2)	1404–1469 (3)	1206–1271 (3)	1347–1410 (4)	1346–1409 (2)	1349–1412 (4)
*cox1*	J	1406–2948 (−8)	1467–3009 (−8)	1462–3004 (−8)	1264–2806 (−8)	1403–2945 (−8)	1402–2944 (−9)	1405–2949 (−8)
*trnL2(UUR)*	J	2949–3013	3010–3074	3005–3069	2807–2871	2946–3010	2945–3009	2952–3016 (2)
*cox2*	J	3014–3701	3075–3762	3070–3753	2872–3559	3011–3698	3010–3697	3017–3704
*trnK*	J	3702–3772	3763–3833	3758–3828 (4)	3560–3630	3699–3769	3698–3768	3705–3775
*trnD*	J	3772–3836 (−1)	3834–3899	3829–3893	3631–3695	3769–3833 (−1)	3768–3823 (−1)	3776–3840 (−1)
*atp8*	J	3837–3998	3900–4061	3894–4055	3696–3857	3834–3995	3833–3994 (9)	3841–4002
*atp6*	J	3989–4660 (−10)	4052–4723 (−10)	4046–4717 (−10)	3848–4519 (−10)	3986–4657 (−10)	3985–4656 (−10)	3993–4664 (−10)
*cox3*	J	4660–5442 (−1)	4723–5505 (−1)	4717–5499 (−1)	4519–5301 (−1)	4657–5437 (−1)	4656–5436 (−1)	4664–5444 (−1)
*trnG*	J	5444–5508 (1)	5521–5584 (15)	5515–5578 (15)	5317–5380 (15)	5438–5500	5437–5500	5445–5507
*nad3*	J	5506–5862 (−3)	5582–5938 (−3)	5576–5932 (−3)	5378–5734 (−3)	5498–5854 (−3)	5498–5854 (−3)	5505–5861 (−3)
*trnA*	J	5866–5930 (3)	5937–6001 (−2)	5931–5995 (−2)	5733–5797 (−2)	5853–5918 (−2)	5853–5917 (−2)	5860–5925 (−2)
*trnR*	J	5930–5992 (−1)	6001–6067 (−1)	5995–6060 (−1)	5797–5863 (−1)	5918–5981 (−1)	5917–5980 (−1)	5925–5989 (−1)
*trnN*	J	5993–5958	6068–6132	6061–6126	5864–5929	5981–6045 (−1)	5980–6044 (−1)	5990–6056 (−1)
*trnS1(AGN)*	J	6059–6116	6133–6191	6127–6185	5930–5988	6046–6102	6045–6101	6057–6114
*trnE*	J	6120–6181 (3)	6194–6255 (2)	6188–6249 (2)	5991–6052 (2)	6103–6164	6102–6163	6115–6176
*trnF*	N	6180–6243 (−2)	6254–6318 (−2)	6248–6312 (−2)	6051–6115 (−2)	6163–6225 (−2)	6162–6224 (−2)	6175–6240 (−2)
*nad5*	N	6244–7954	6319–8029	6313–8023	6116–7826	6226–7936	6225–7935	6241–7951
*trnH*	N	7955–8016	8030–8094	8024–8088	7827–7891	7937–8000	7936–7999	7949–8011
*nad4*	N	8017–9349	8095–9427	8089–9421	7892–9224	8001–9333	8000–9332	8012–9344
*nad4L*	N	9343–9630 (−7)	9421–9708 (−7)	9415–9702 (−7)	9218–9505 (−7)	9327–9614 (−7)	9326–9613 (−7)	9338–9625 (−5)
*trnT*	J	9633–9696 (2)	9711–9773 (−2)	9705–9767 (2)	9508–9570 (2)	9617–9680 (2)	9616–9678 (2)	9628–9690 (2)
*trnP*	N	9697–9759	9774–9837	9768–9831	9571–9633	9681–9744	9679–9742	9691–9755
*nad6*	J	9762–10,256 (2)	9840–10,331 (2)	9834–10,325 (2)	9636–10,127 (2)	9747–10,238 (2)	9745–10,236 (2)	9758–10,249 (2)
*cob*	J	10,256–11,393 (−1)	10,331–11,470 (−1)	10,325–11,464 (−1)	10,127–11,266 (−1)	10,238–11,377 (−1)	10,236–11,375 (−1)	10,249–11,385 (−1)
*trnS2(UCN)*	J	113,394–11,461	11,469–11,536 (−2)	11,463–11,530 (−2)	11,265–11,332 (−2)	11,376–11,443 (−2)	11,374–11,441 (−2)	11,384–11,451 (−2)
*nad1*	N	11,479–12,429 (17)	11,718–12,668 (181)	11,653–12,603 (123)	11,443–12,393 (110)	11,461–12,411 (17)	11,459–12,409 (17)	11,469–12,419 (17)
*trnL1(CUN)*	N	12,430–12,493	12,669–12,733	12,604–12,667	12,394–12,457	12,412–12,476	12,410–12,473	12,420–12,482
*rrnL*	N	12,494–13,767	12,734–14,014	12,668–13,943	12,458–13,735	12,477–13,757	12,474–13,750	12,483–13,759
*trnV*	N	13,768–13,836	14,015–14,083	13,944–14,012	13,736–13,804	13,758–13,826	13,751–138,181	13,760–13,828
*rrnS*	N	13,837–14,619	14,084–14,876	14,013–14,798	13,805–14,370	13,827–14,615	13,819–14,608	13,829–14,618
control region		14,620–15,758	14,877–16,003	14,798–15,923		14,616–15,816	14,609–15,691	14,619–15,633

^a^Majority (J), Minority (N) coding strands

Numbers in parenthesis represent the intergenic nucleotides, negative values refer to overlapping nucleotides

### A + T content and codon usage

**Fig. 2:**
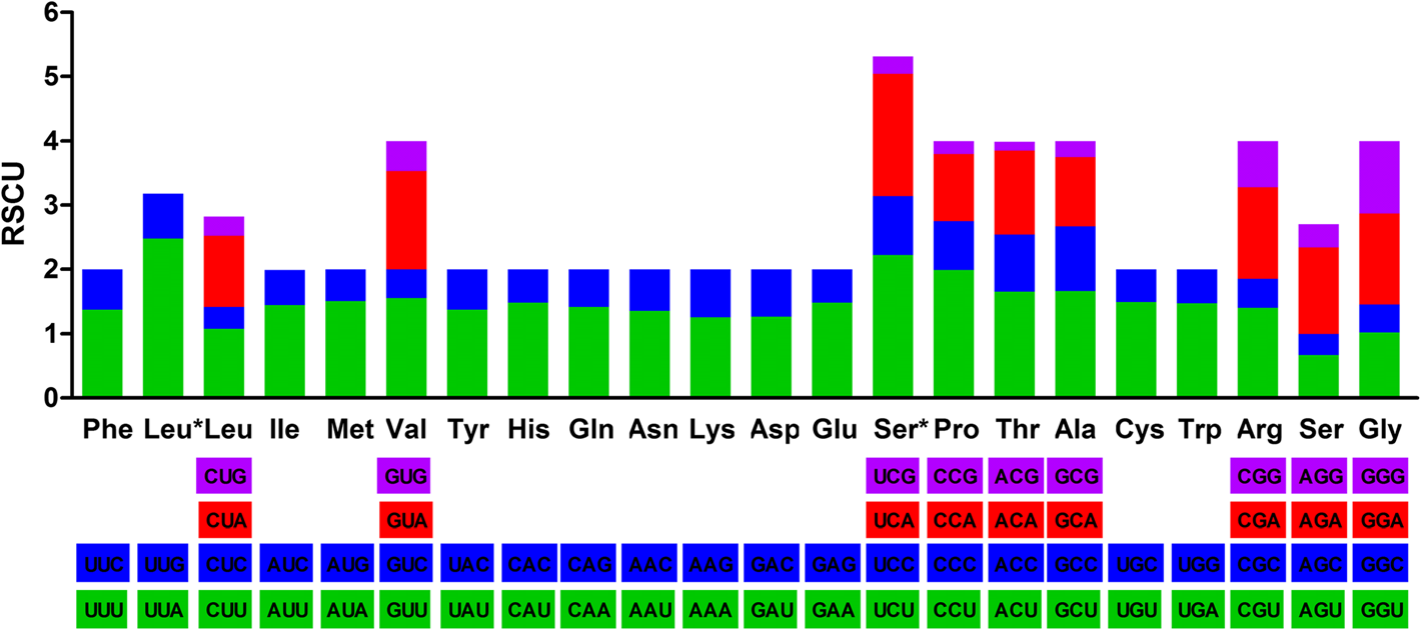
Relative synonymous codon usage (RSCU) in mitochondrial genomes of seven meloids. Average 3701 codons for meloids were analyzed, excluding stop codons. The RSCUs are averages over all seven meloid taxa. Codon families are provided on the x-axis. Leu, Leu*, Ser, and Ser* indicate *trnL1 (CUN)*, *trnL2 (UUR)*, *trnS1 (AGN)*, and *trnS2 (UCN)*, respectively

The overall A + T contents of the seven meloid mitogenomes range from 67.53% to 71.94% (Table 1), and such an A + T bias is reflected in the codon frequencies of these mitogenomes (Fig. 2). Relatively synonymous codon usages (RSCU) were calculated over all seven meloid species, excluding stop codons (Additional file 8: Table S8). The RSCU demonstrate that codons with A or T in the third position are always overused as compared to other synonymous codons. Additionally, codons TTT (Phe), TTA (Leu), ATT (Ile), and ATA (Met) are the four most frequently used codons in these meloid mitogenomes. These codons are all comprised of A or T nucleotides, which is indicative of the biased usage of A and T nucleotides in the meloid mitochondrial PCGs.

### Evolutionary rates of PCGs

All available mitogenomes were used to assess the evolutionary rate of PCGs for Meloidae. The variable sites, nucleotide diversity (π), and the ratio of non-synonymous substitution (Ka) to synonymous substitution (Ks) were calculated for each PCG (Table 3). *Nad6* and *atp8* genes exhibit the highest level of nucleotide diversities, whereas *cox1* gene is the most conserved. The Ka/Ks value is correlated with the nucleotide diversity, with the highest level in *nad6* and *atp8* and the lowest in *cox1*. Notably, the Ka/Ks ratio for every PCG is lower than 1, indicating that all PCGs are evolving under the purifying selection.

**Table 3:** Evolutionary rates of mitochondrial PCGs among meloid species

PCG	Length (bp)	Variable sites	π	Ka/Ks
*atp6*	669	298	0.2590	0.13
*atp8*	159	97	0.4473	0.38
*cox1*	1542	569	0.2086	0.06
*cox2*	687	306	0.2539	0.09
*cox3*	780	336	0.2449	0.10
*cob*	1146	506	0.2663	0.12
*nad1*	951	445	0.2583	0.19
*nad2*	1011	564	0.3649	0.28
*nad3*	354	196	0.3474	0.22
*nad4*	1332	661	0.2903	0.23
*nad4l*	285	130	0.2727	0.17
*nad5*	1710	837	0.2884	0.21
*nad6*	492	312	0.4489	0.37

*π* nucleotide diversity, *Ka/Ks* the ratio of non-synonymous substitution (Ka) to synonymous substitution (Ks), *ts/tv* transition to transversion ratio

### Comparison of tRNA secondary structures

**Fig. 3:**
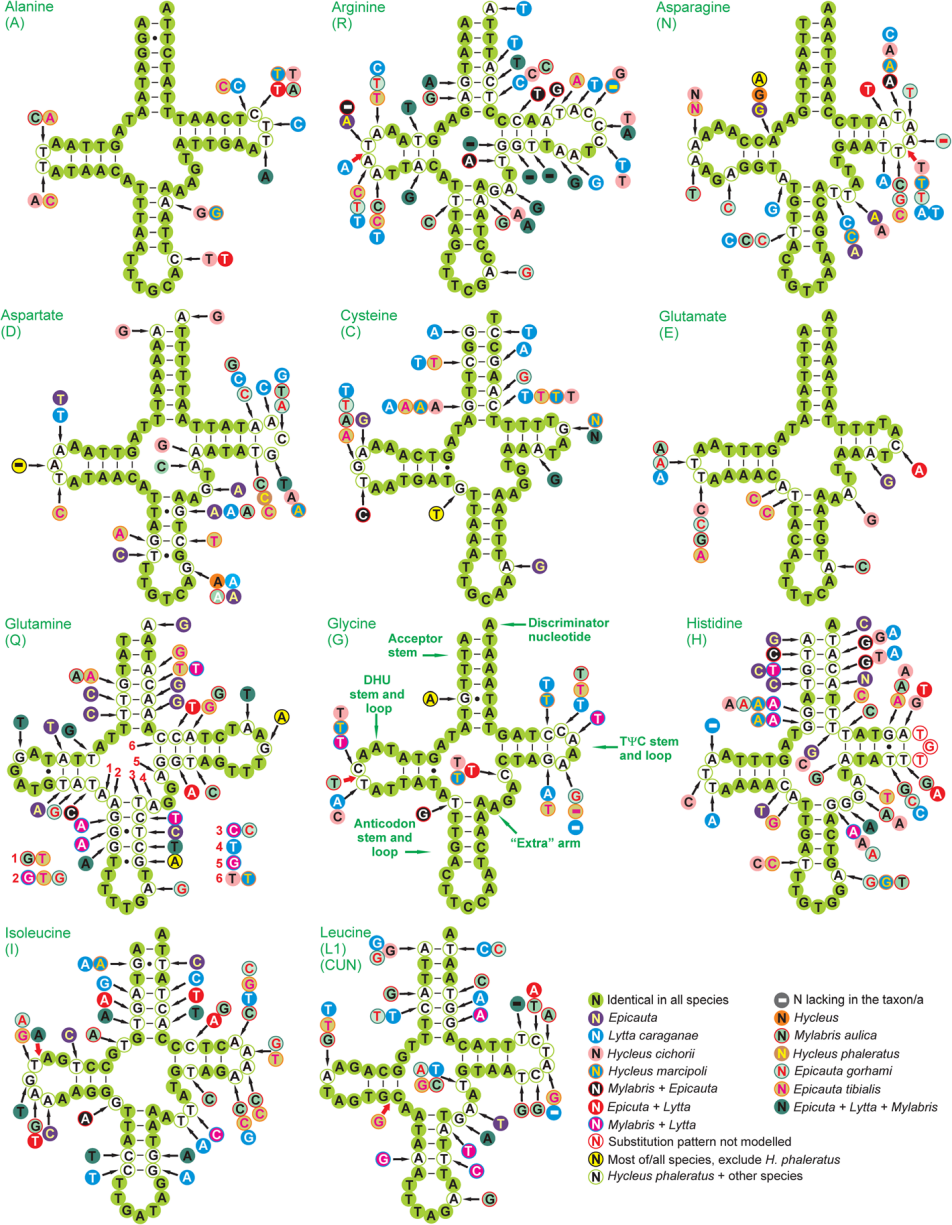
Secondary structure of tRNAs (*trnA-trnL1*) in meloid mitogenomes. The nucleotide substitution pattern for each tRNA was modeled using as reference the structure determined for *Hycleus phaleratus*

**Fig. 4:**
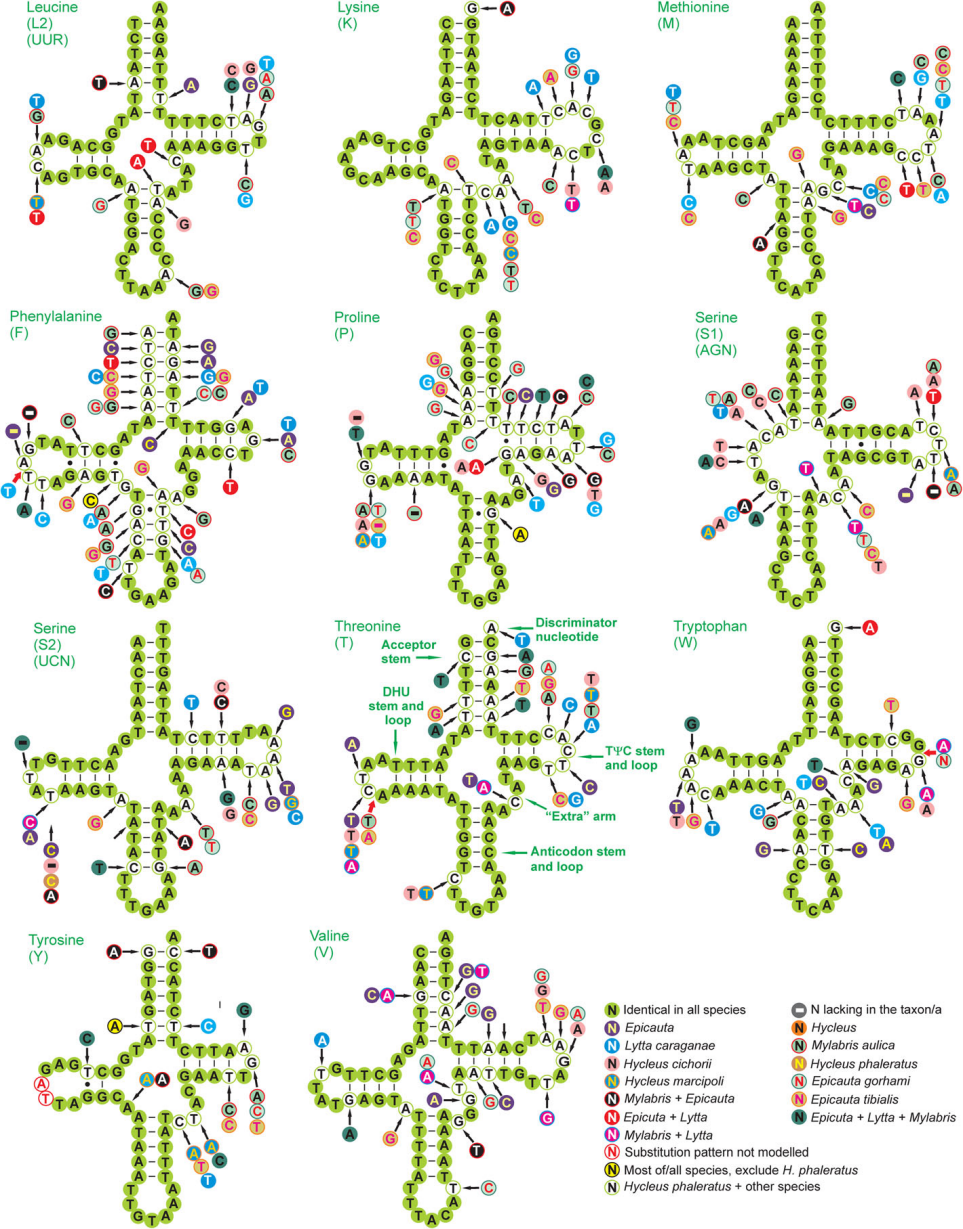
Secondary structure of tRNAs (*trnL2-trnV*) in meloid mitogenomes. The nucleotide substitution pattern for each tRNA was modeled using as reference the structure determined for *Hycleus phaleratus*

All seven meloid mitogenomes encode 14 tRNAs on the J strand and 8 on the N strand (Fig. 1, Table 2). The comparative results of tRNA secondary structures are provided in Figs. 3 and 4. All tRNAs could be folded into the typical clover-leaf structure, except *trnS1 (AGN)* lacks a dihydrouridine (DHU) arm, which is replaced by a simple loop (Fig. 4).

The nucleotide conservation of tRNAs is markedly J strand-biased, *trnA*, *trnE*, *trnG* and *trnK*, which have the highest percentage of identical nucleotides, are all located on the J strand. The tRNAs on the N strand (i.e., *trnQ*, *trnH*, *trnF*) and close to the control regions (i.e., *trnI* and *trnQ*) have high level of nucleotide variation (Additional file 9: Table S9).

### Intergenic spacers

**Fig. 5:**
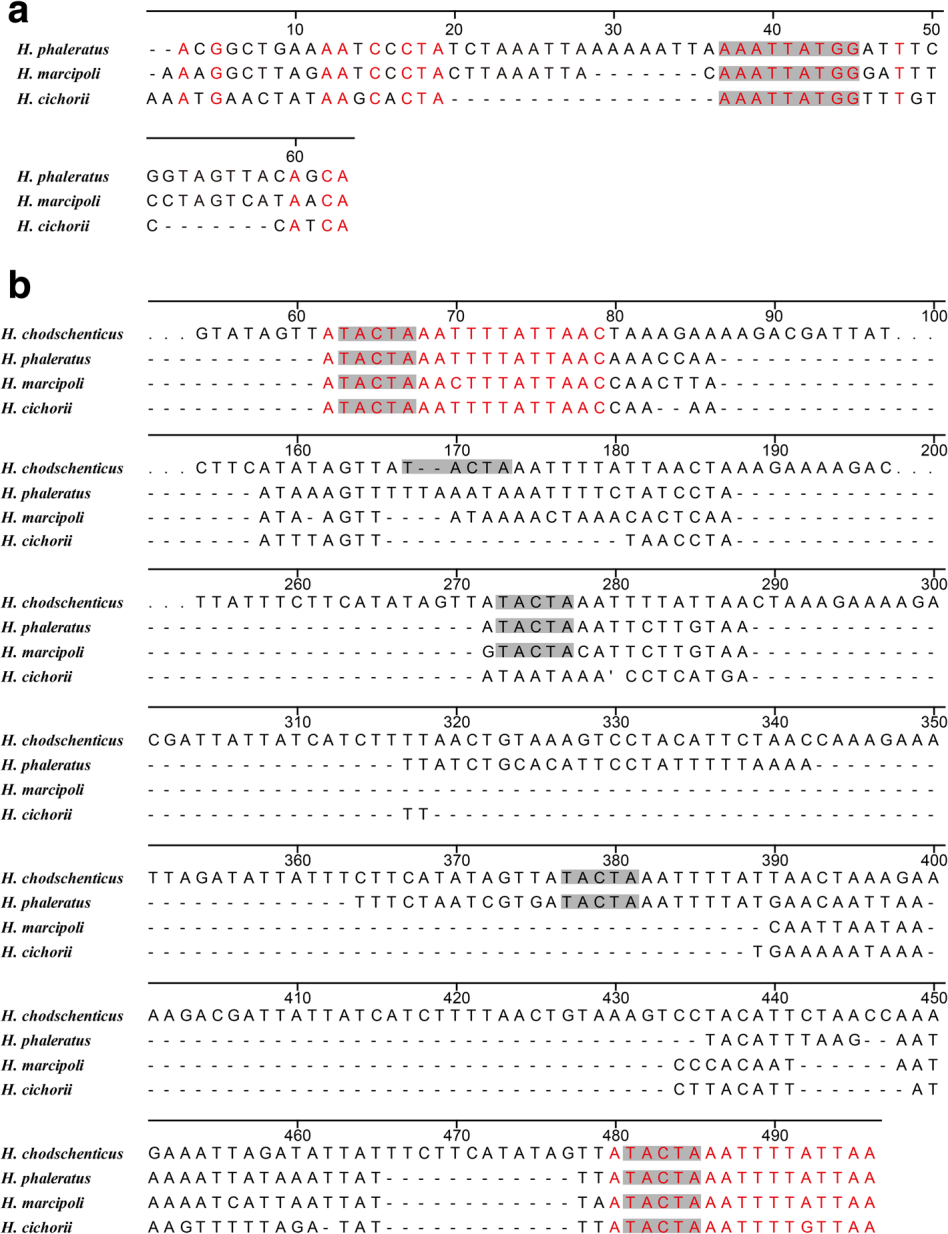
Alignments of the intergenic spacers in *Hycleus* mitochondrial genomes. **a** IGS1 located between *trnW* and *trnC*. The nucleotides in red refer to consistent ones in three *Hycleus* species. The shaded nucleotides indicate the congruent motif (AAATTATGG) found in *Hycleus* insects. - refers to gap (**b**) IGS2 located between *trnS2* and *nad1*. The shaded nucleotides indicate the conserved pentanucleotide motif (TACTA). The nucleotides in red indicate the 18 bp conservative sequence ATACTAAAYTTTRTTAAC. - refer to gaps, dots refer to omitted nucleotides

**Fig. 6:**
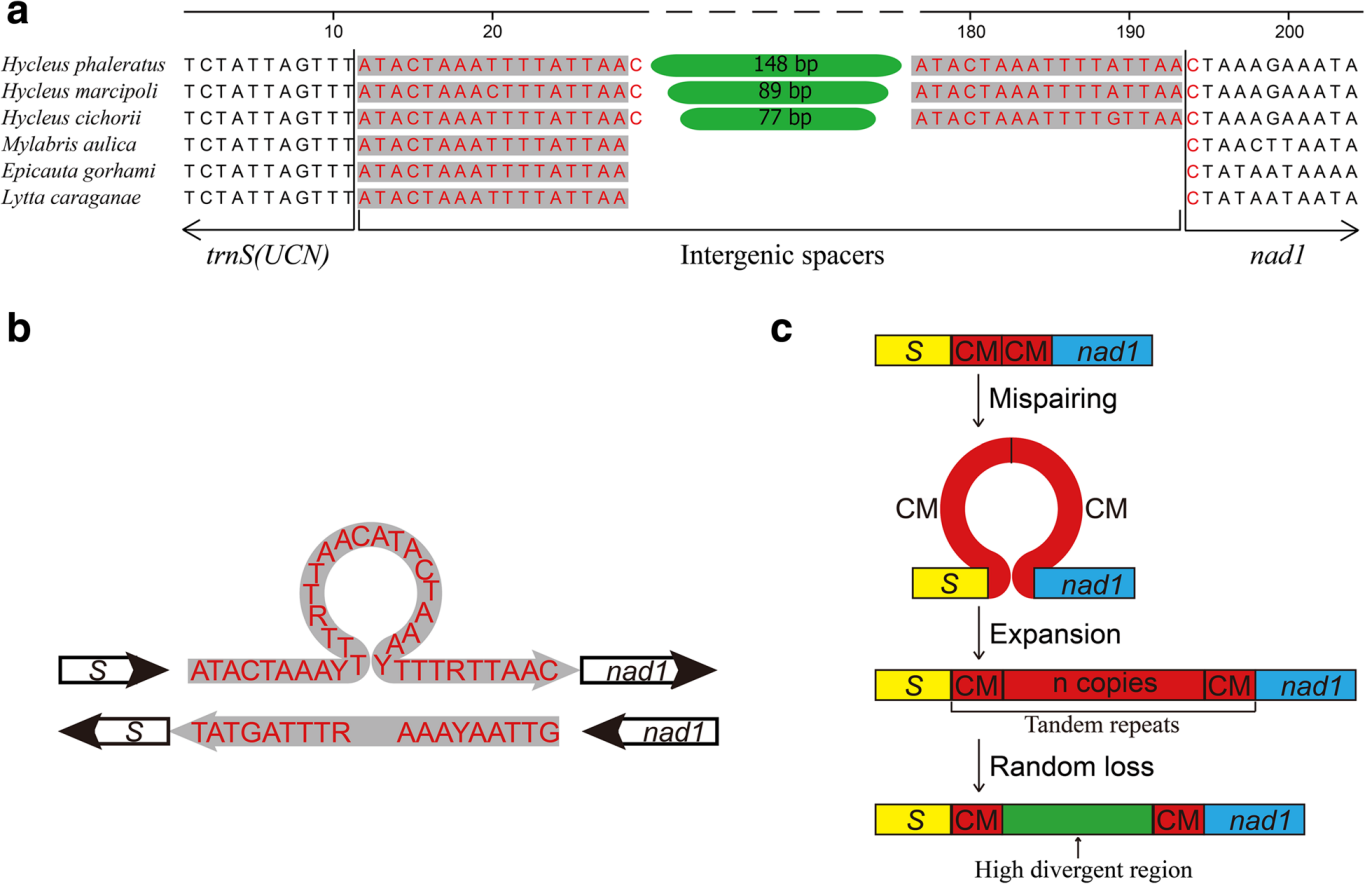
**a** Organizations of the intergenic spacers between *trnS2 (UCN)* and *nad1* in mitogenomes of all meloids. **b** Putative mispairing involves replicating DNA strands of *Hycleus* mitogenomes. **c** The proposed mechanism of IGS2 in *Hycleus* mitogenomes. The CS indicates the 18 bp conservative sequence ATACTAAAYTTTRTTAAC

The mitogenomes of *E. gorhami*, *E. tibialis*, and *L. caraganae* contain four intergenic spacers with a total length of 23, 25, 26 bp, respectively, while the *M. aulica* has six intergenic spacers with a total length of 28 bp (Table 2). Unexpectedly, *H. phaleratus*, *H. marcipoli*, and *H. cichorii* contain 7, 8, 8 intergenic spacers with a total length of 266, 203, 175 bp, respectively (Table 2), including two large intergenic spacers (>30 bp). IGS1 is located between *trnW* and *trnC* with a length of 62, 56, 39 bp in *H. phaleratus*, *H. marcipoli*, and *H. cichorii*, respectively (Table 2). The IGS1 sequences exhibit relatively high similarity among the three *Hycleus* species, and a 9 bp long congruent motif AAATTATGG was detected in the three *Hycleus* species (Fig. 5a). IGS2 is located between *trnS2* and *nad1* with a length of 181, 123, and 110 bp in *H. phaleratus*, *H. marcipoli*, and *H. cichorii*, respectively (Table 2). The alignment of IGS2s among all sequenced *Hycleus* mitogenomes, including the recently published sequence of *H. chodschenticus* [31], shows that a pentanucleotide motif (TACTA) exists in these *Hycleus* species. Furthermore, *H. chodschenticus*, *H. phaleratus*, *H. marcipoli*, and *H. cichorii* include five, four, three, and two repeats (respectively) of this motif (Fig. 5b). The organization of IGS2 indicates that these four *Hycleus* species contain two copies of an 18 bp conserved sequence (ATACTAAAYTTTRTTAAC) in both ends of IGS2 (Fig. 5b), but other meloid beetles have only one (Fig. 6a).

### Phylogenetic relationships

**Fig. 7:**
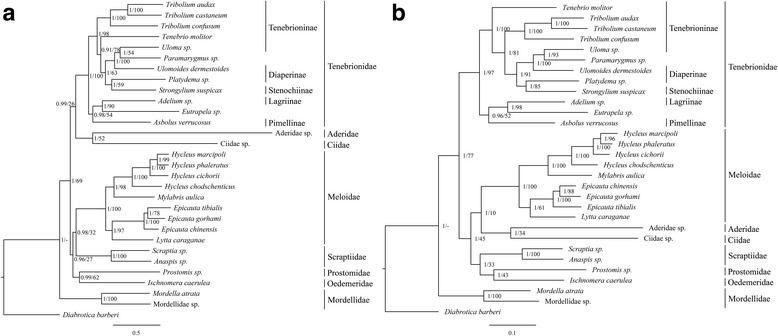
The phylogenetic tree of 16 species from superfamily Tenebrionoidea based on the nucleotide dataset (**a**) and the amino acids dataset (**b**) of 13 mitochondrial protein-coding genes, inferred from Bayesian inference and maximum likelihood. The numbers abutting branches refer to Bayesian posterior probabilities (left) and ML bootstraps (right), − not recovered. Branch lengths and topology are from BI analyses. The *Diabrotica barberi* (Coleoptera: Chrysomelidae) was employed to root the trees as outgroup

We carried out MrBayes, RAxML, and PhyloBayes analyses based on nucleotide and amino acid datasets to determine the influence of different datasets and analytical methods on tree topology and node reliability. Bayesian Inference (BI) and Maximum Likelihood (ML) analyses used the same datasets to generate congruent tree topologies. BI trees had higher node support values than ML trees (Fig. 7). PhyloBayes analyses generated different tree topologies with polytomies (Additional file 10: Figure S1). Four tree topologies derived from our six phylogenetic trees are consistent with the monophylies of Meloidae and Tenebrionidae, and the basal position of Mordellidae. Differences are present in the inter-family relationships of Aderidae, Ciidae, Oedemeridae, and Prostomidae.

Within Meloidae, *L. caraganae* is sister taxon to the species belonging to the genus *Epicauta*. The Meloidae family results monophyletic and receives maximum statistical support. In Tenebrionidae, the Tenebrioninae and Diaperinae are never recovered as monophyletic groups (Fig. 7, Additional file 10: Figure S1).

## Discussion

### General features

All seven mitochondrial genomic arrangements share the ancestral type for insects [26, 38], as is reported in the published meloid mitogenomes [31, 37]. The nucleotide composition of mitochondrial genomes for meloids also corresponds well to the A + T bias generally observed in insect mitogenomes.

All mitogenomes of the seven meloids have incomplete stop codons, which have been described in many other insect species [39, 40]. It has been demonstrated that incomplete stop codons can produce functional stop codons in polycistronic transcription cleavage and polyadenylation processes [9]. Remarkably, the *cox3* genes of *Lytta* and *Epicauta* species possess the same incomplete stop codon, while *Hycleus* and *Mylabris* beetles utilize complete terminators (Additional files 1, 2, 3, 4, 5, 6 and 7: Tables S1–S7). The similar preference for the adoption of stop codons seems to suggest that the genus *Lytta* is more closely related to *Epicauta* than the two other genera, and this relationship was confirmed by phylogenetic results.

The evolutionary rates of all mitochondrial PCGs indicate that their evolution is based on purifying selection (Table 3), as is reported in other insects [41, 42]. However, the cytochrome oxidase subunits (*cox1*, *cox2*, and *cox3*) and cytochrome b (*cob*) have lower Ka/Ks ratios than ATPase subunits (*atp8* and *atp6*) and NADH dehydrogenase subunits (*nad1–6* and *4 L*). The nucleotide diversity also shows *cox* and *cob* genes are obviously more conserved than *atp* and *nad* genes. This phenomenon indicates that the various functional genes in the mitochondria of meloids underwent different selection pressures during evolution. Furthermore, the *cox1* has the slowest evolutionary rate, demonstrating that functional constraints are more powerful for this gene than positive selection.

The absence of a DHU arm of *trnS1* commonly exists in many metazoan mitogenomes, including insects [30, 43, 44]. However, this tRNA (missing DHU arm) was often suggested by authors to be functional [45, 46]. Another unusual feature is the use of TCT as the *trnS1* anticodon in meloids, although most arthropods adopt a GCT anticodon in *trnS1*. This exceptional *trnS1* anticodon was also found in many other beetles [5, 30, 47].

Some mismatched pairs in stems of tRNAs (e.g., T-T in the DHU stem of *trnQ* and in anticodon stem of *trnK*; C-T in the TΨC stem of *trnI*; A-C in anticodon stem of *trnL2 (UUR)*; A-G in acceptor stem of *trnW*), are common in insect mitogenomes and can be corrected through editing processes or may represent unusual pairings [44]. It was not possible to model the substitution pattern of the TΨC loop in *trnH* (Fig. 3) because of the high variation among orthologous sequences. The increasing variation usually accompanies more compensatory base changes in stems, resulting in the tRNA more or less not conserved (Additional file 9: Table S9).

The ends of rRNA genes of meloid mitogenomes were assumed to extend to the boundaries of flanking genes because it is impossible to accurately determine by DNA sequence alone [48]. Consequently, *rrnL* was assumed to fill up the blank between *trnV* and *trnL1 (CUN)* (Fig. 1), but the boundary between the *rrnS* and the putative control region was defined by the alignment with homologous sequences [49].

The control region in the insect mitogenome is equivalent to the control region of vertebrate mitogenomes, which contains the origin sites for transcription and replication [50, 51]. The six complete mitogenomes include a poly-T stretch (15 bp) that was suggested to function as a possible recognition site for the initiation of replication of the mitochondrial DNA N strand [50]. Like other Coleopteran mitogenomes, the control regions of meloids also exhibit the highest A + T content in the whole mitogenome. This region is unlikely to be more variable than protein-coding genes due to such high A + T content and consequently limits its usefulness as a molecular marker [52].

### Intergenic spacers

All newly sequenced mitochondrial genomic arrangements share the ancestral type for insects without rearrangement, but possess large non-coding regions (except the control region) in some lineages. The intergenic spacers in the mitogenomes of *E. tibialis*, *E. gorhami* and *L. caraganae* are similar to those in *E. chinensis* mitogenome [37]. The total length of *M. aulica*’s intergenic spacers is not significantly different from the former four meloids, but its genome does contain more intergenic spacers (Table 2). Unexpectedly, the whole lengths of intergenic spacers in the three *Hycleus* mitogenomes are much longer than those of other meloids. The most remarkable feature is the presence of two IGSs in the mitogenomes of three *Hycleus* species. A 494-bp long intergenic spacer was also reported in the recently published mitogenome of *H. chodschenticus* [31]. Consequently, the total lengths of known *Hycleus* mitogenomes are longer than those of other meloid mitogenomes, but predominantly due to the presence of IGSs rather than the lengths of genes or control regions.

The mitochondrial genome typically displays exceptional economy of organization, evidenced by lack of introns, few intergenic spacers, incomplete stop codons and even overlapping genes [9]. However, the large IGSs in mitochondrial genomes were observed in some Hymenopteran [11, 13], Hemipteran [15, 17] Dictyopteran [18] and Coleopteran [5, 30] insects. Previously reported IGSs contain tandem repeat units (in *Pyrocoelia rufa* and *Evania appendigaster*) [5, 11], or additional origin of replication [10] and similar to it [15]. The IGS in Reduviidae bugs have unidentified open reading frames encoding 103 or 104 amino acids but without blast similarity [15, 17]. In contrast, the two IGSs in *Hycleus* species have no significant similarity with other genes of their mitogenomes and lack open reading frames or tandem repeats. Nevertheless, the IGS2 of all studied *Hycleus* species contain discontinuous repeats of a 5 bp consensus motif (TACTA) (Fig. 5b). This motif was also found in many other Coleopteran insects [30], similar to the 7 bp motif ‘ATACTAA’ conserved in Lepidoptera [6] and the hexanucleotide motif ‘THACWW’ in Hymenoptera [11]. The pentanucleotide motif was suggested as the possible binding site of a transcription termination peptide (mtTERM), as its position signifies the end of the J strand coding region in the circular mitochondrial DNA [38]. However, we do not know the function of this discontinuous repeat.

The discontinuous repeats and the 18 bp long consensus sequence in both ends of IGS2 within all studied *Hycleus* species (Fig. 6a) suggest that the slipped-strand mispairing [53] may be the evolutionary mechanism of this IGS. According to this theory, mispairing involves dissociation of replicating DNA strands and then misaligned reassociation (Fig. 6b), following replication or repair lead to insertions of several repeat units. Formed tandem repeat experiences random loss and/or point mutation, only the repeat units in both ends are completely retained and the residues form the IGS2 (Fig. 6c). Although we are not absolutely certain about our assumption due to the highly divergent region, the fragmented repeat units in highly divergent region (Fig. 5b) and complete repeat units at both ends (Fig. 6a) suggest that the slipped-strand mispairing is the most convincing mechanism for IGS2.

**Fig. 8:**
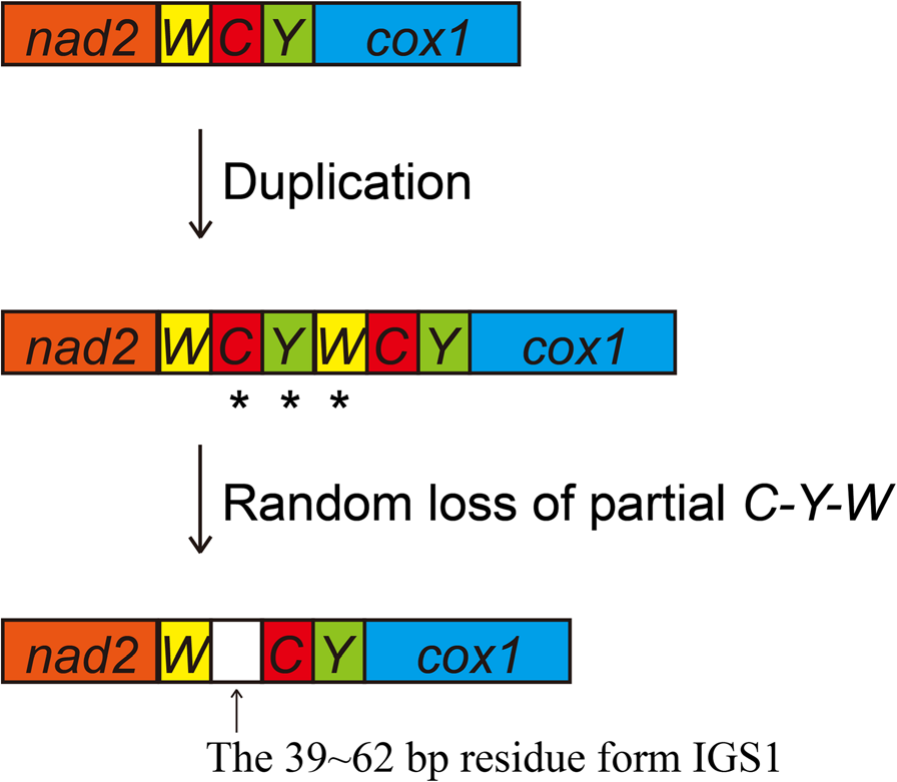
Putative mechanism of IGS1 in mitogenomes of *Hycleus* species under the duplication/random loss model. The random losses of partial genes are marked with *

The IGS between *trnW* and *trnC* was only previously reported in *Trachypachus holmbergi* [30], while no IGS (>30 bp) at this position has yet been found in the mitogenomes for Meloidae. Although *H. chodschenticus* has 3-bp intergenic spacer at the same position [31], it is too usual as many short intergenic spacers to be considered as IGS1. Therefore, the evolutionary mechanism of IGS1 in the three *Hycleus* species in present study is different from *H. chodschenticus*. The 9-bp consistent motif and the relatively high similarity among the three *Hycleus* species in the present study (Fig. 5) suggest that they have the mutual mechanism of IGS1. The duplication/random loss model [1, 54] may account for the IGS1. We speculate that tandem duplication of *trnW*-*trnC*-*trnY* is caused by some error in DNA replication, followed by random loss of partial duplicated genes, and then the residues form the IGS1 (Fig. 8). This model was also proposed as the mechanism for rearrangements in some Hemipteran [17] and Mantodea mitogenomes [19], and the IGSs in *Blaptica dubia* [18]. It is possible that the duplication/random loss event of *Hycleus* beetles occurred relatively early and many nucleotides were deleted during the random loss period. Consequently, the residual IGS1 has low similarity with the original tRNAs, and the IGS1 is somewhat not conserved in *Hycleus*.

Similar to the three *Hycleus* species in the present study, *H. chodschenticus* also possesses the IGS between *trnS2* and *nad1* [31]. Although intergenic spacers with approximately 20 bp at this position are common in Coleopteran mitogenomes, no large intergenic spacers have been found within other Coleopteran lineages to date. Based on the current knowledge of IGS2 across Coleoptera, it is reasonable to assume that the emergence of IGS2 in *Hycleus* mitogenomes might be a special evolutionary mutation. It is difficult to identify the closely related genera *Hycleus* and *Mylabris* since they share very similar morphological characteristics. However, IGS2 might be a potential marker to distinguish *Hycleus* from its closely related and indistinguishable genera with respect to the sizeable intergenic spacer that exists in all studied *Hycleus* species but is absent in other genera,. Although we are unable to adequately confirm that IGS2 exists in all *Hycleus* species due to limited samples, it provides a new candidate for molecular identification of this genus. Variations in the quantity, location and sequence of intergenic spacers might also be a valuable marker for phylogenetic and evolutionary studies at lower taxonomic levels, if these details of more taxa were obtained in future.

### Phylogeny

Phylogenetic studies indicated that different datasets and inference methods influence the tree topology of Coleoptera [31, 55]. Our phylogenetic results showed that tree topologies are sensitive to datasets rather than inference methods, since the different inference methods with the same datasets generated consistent tree topologies. The heterogeneous-site model in PhyloBayes was suggested as being more reliable for phylogenetic inferences within Coleoptera [31, 55]. Our Bayesian analyses under the heterogeneous-site model are unable to resolve phylogenetic relationships within Tenebrionoidea, but perhaps this is due to insufficient taxa. Although the nucleotide dataset of PCGs was proposed as better for phylogenetic analyses at superfamily level [31], we could not assess the quality of different datasets because some key lineages share the same tree topologies. However, nodal support values are sensitive to inference methods. For the same datasets, ML trees had significantly lower support values than BI trees, especially at several nodes that involved the family-level relationships of Aderidae, Ciidae, Scraptiidae (Fig. 7). This is consistent with previous phylogenetic studies using mitogenomes [14, 31, 37].

Phylogenetic relationships within Tenebrionoidea are ambiguous. The inter-family relationships are also uncertain, especially for Aderidae, Ciidae, Oedemerdae, and Prostomedae, which are respectively represented by only one taxon. However, all tree topologies well recover the monophyly of Tenebrionidae, Meloidae, and Mordellidae (Fig. 7, Additional file 10: Figure S1). Tenebrioninae and Diaperinae are never recovered as monophyletic groups, as is reported by Gunter et al. [56]. To date, no phylogeny has successfully resolved the interfamilial relationships between tenebrionoids, either by using morphological or molecular characteristics. The comprehensive phylogeny of Coleoptera [57, 58] and the largest molecular phylogeny of Tenebrionoidea [56] were unable to recover strong support or definitively infer the phylogenetic relationships within the superfamilies. In contrast to these phylogenetic studies based on several genes, our phylogenetic inferences may be hindered by lack of across taxon sampling rather than dataset validity.

The genus *Lytta* is the sister group to *Epicauta* rather than grouped with *Mylabris* within Meloidae (Fig. 7). The placement of *Lytta* and whether *Lytta* is more closely related to *Mylabris* or *Epicauta* could not be inferred from previous molecular phylogeny of the family Meloidae based on 16S rRNA and ITS2 [32]. We confirmed this relationship by multiple inference approaches based on both nucleotide and amino acid sequences of 13 mitochondrial PCGs. Considering the high diversity and rapid radiations of insects [59, 60], mitochondrial genomes could be a better approach to resolve intractable phylogenetic relationships due to its relatively rapid rate of mutation and purely maternal inheritance [3, 61]. Consequently, we believe our conclusion that the genus *Lytta* is more closely related to *Epicauta* than *Mylabris* or *Hycleus* is reliable because it is based on complete mitogenomes and the preference of stop codon usage.

The data collected thus far regarding meloid mitogenomes could not resolve the phylogenetic relationships within Meloidae. In fact, no phylogeny of Meloidae based on either morphological or molecular characteristics has been able to successfully resolve the relationships at genera and species levels. Taxon sampling is known to be one of the most significant determinants of accurate phylogenetic inferences, particularly in species rich lineages [62, 63]. Considering the diversity of the family Meloidae and the limitation of the present molecular information, more conclusive phylogenetic results will be achievable as bio-information becomes increasingly available. This study will assist with these more conclusive phylogenetic results and future studies on taxonomy, phylogeny and systematics of Meloidae insects.

## Conclusions

Our study presents the mitochondrial genomes of seven meloid beetles. All complete mitogenomes of meloids retain the typical gene content and organization of the ancestor. The evolutionary rates of all PCGs in the studied Meloidae indicate that their evolution is according to purifying selection. The comparison of tRNA secondary structures exhibit diverse substitution patterns in Meloidae. Two large intergenic spacers exist in the three studied *Hycleus* mitogenomes, and the sequence and structure of the two IGSs contributed to our conclusion regarding their possible evolutionary mechanisms. The phylogenetic results inferred from mitochondrial genomes support that the genus *Lytta* is more closely related to *Epicauta* than to *Mylabris*. Although data collected thus far could not resolve the phylogenetic relationships within Meloidae, this study will assist in future mapping of the Meloidae phylogeny.

## Methods

### Samples collection and DNA extraction

The specimens of the seven meloid species used for this study are listed in Table 1, with locality data. The fresh materials were immediately preserved in 100% ethanol and stored in a − 20 °C refrigerator. Total genomic DNA was extracted from a frozen adult using Tianamp Genomic DNA kit.

### PCR amplification and sequencing

All mitochondrial genomes of these collected meloid species were generated by amplifications of nine overlapping PCR fragments. Eight fragments were amplified using common primers for all seven species designed from the aligned *E. chinensis* mitogenome (GenBank accession number KP692789) [37], only the primers of the control region were specifically designed for each species. Details of primers are given in Additional file 11: Table S10. The PCR was performed with Vazyme Taq DNA Polymerase (Mg^2+^ Plus Buffer) and carried out on a PTC-100 thermal cycler (BioRad, Hercules, CA). PCR conditions used were: 5 min denaturation at 94 °C; 35 cycles of 30s at 94 °C, 30s at 49–56 °C and 1–3 min (1 min/Kbp) at 72 °C; followed by 10 min extension hold at 72 °C. The PCR products were sequenced on an ABI PRISM 3730 DNA sequencer by Tsingke Biotechnology Company with primers walking on both strands.

### Genome assembly and annotation

Sequences from overlapping fragments were assembled with the neighboring fragments using SeqMan program included in the Lasergene software package (DNAStar Inc., Madison, Wisc.). Protein-coding regions were identified by ORF Finder from the NCBI website with invertebrate mitochondrial genetic codes, and compared with published mitochondrial sequences by using MEGA 6.0 [64]. Most of the tRNA genes were identified using tRNAscan-SE 1.21 [65] with invertebrate genetic codon predictors; however, the *trnS1* was predicted by alignments with other homologous genes because of its lack of dihydrouridine (DHU) arm. The rRNA gene boundaries were interpreted as the end of a bounding tRNA gene and alignment of sequences with homologous regions of known Coleopteran mitogenomes. The control regions were assumed between *rrnS* and *trnI* within all meloid mitochondrial genomes. The A + T contents, relative synonymous codon usage values, and evolutionary rates (number of variable sites, nucleotide diversity, and Ka/Ks ratios) for each PCG were calculated via MEGA 6.0 [64].

### Phylogenetic analysis

Phylogenetic analyses were assessed using 29 Tenebrionoidea species representing 8 families, with the Chrysomelid *Diabrotica barberi* (GenBank accession number NC_022935.1) [8] employed as the outgroup. Species’ PCGs were extracted according to GenBank annotations by using GenScalpel [66]. All these nucleotide and amino acid sequences were aligned using MUSCLE [67] with the default setting. Gaps and ambiguous sites were removed from the protein alignment to generate a 10,356-bp nucleotide dataset and a corresponding amino acid dataset (3452 amino acids). The best partitioning schemes and corresponding evolutionary models were selected by PartitionFinder 1.1.1 [68] with 13 partitions defined by genes. We set the model selection as Bayesian information criterion (BIC), unlinked branch lengths, and greedy search algorithm to estimate the best fitting schemes. The best-fit partitioning schemes and corresponding models are shown in Additional file 12: Table S11.

Phylogenetic analyses with site-homogeneous model were conducted to reconstruct the phylogenetic trees of superfamily Tenebrionoidea by using Bayesian inference (BI) and maximum likelihood (ML) methods. Bayesian phylogenetic analysis was implemented using MrBayes 3.2.2 [69], and ran for 10,000,000 generations sampling per 1000 generations. Bayesian posterior probabilities were estimated using the Markov chain Monte Carlo (MCMC) sampling approach. ML analysis was carried out with RAxML-HPC2 on XSEDE 8.0.24 [70] using 1000 bootstraps to estimate the node support. Bayesian analyses with a site-heterogeneous model were performed using PhyloBayes MPI 1.5a [71] with two MCMC chains run after the removal of constant sites under the CAT-GTR model.

## Additional files

Additional file 1: Table S1. Annotation of the *Mylabris aulica* mitogenome. (DOCX 21 kb)

Additional file 2: Table S2. Annotation of the *Hycleus phaleratus* mitogenome. (DOCX 21 kb)

Additional file 3: Table S3. Annotation of the *Hycleus marcipoli* mitogenome. (DOCX 21 kb)

Additional file 4: Table S4. Annotation of the *Hycleus cichorii* mitogenome. (DOCX 22 kb)

Additional file 5: Table S5. Annotation of the *Epicauta gorhami* mitogenome. (DOCX 21 kb)

Additional file 6: Table S6. Annotation of the *Epicauta tibialis* mitogenome. (DOCX 21 kb)

Additional file 7: Table S7. Annotation of the *Lytta caraganae* mitogenome. (DOCX 21 kb)

Additional file 8: Table S8. Codon usage in mitochondrial genomes of seven meloids. (DOCX 27 kb)

Additional file 9: Table S9. Summary of multiple alignments of tRNAs in meloid mitogenomes. (DOCX 15 kb)

Additional file 10: Figure S1. The phylogenetic tree of 16 species from superfamily Tenebrionoidea based on the nucleotide dataset and the amino acids dataset of 13 mitochondrial protein-coding genes, inferred from PhyloBayes. The numbers abutting branches refer to Bayesian posterior probabilities. The *Diabrotica barberi* (Coleoptera: Chrysomelidae) was employed to root the trees as outgroup. (TIFF 1285 kb)

Additional file 11: Table S10. PCR primers used to amplify the mitochondrial genomes of seven meloids. (DOCX 19 kb)

Additional file 12: Table S11. The best-fit schemes and evolutionary models for two datasets. (DOCX 16 kb)

## Abbreviations

*atp8* and *apt6*: ATPase subunits 8 and 6
BI: Bayesian Inference
*Cob*: Cytochrome b
*cox1*-*cox3*: Cytochrome C oxidase subunit I-III
IGS: Intergenic spacer
Mitogenome: Mitochondrial genome
ML: Maximum Likelyhood
*nad1*-*nad6* and *nad4L*: NADH dehydrogenase subunits 1–6 and 4 L
PCGs: Protein-coding genes
*rrnL*: large (16S) rRNA subunit
*rrnS*: small (12S) rRNA subunit
RSCU: Relatively synonymous codon usages
