# Supplementary material for: Mitochondrial genomes of blister beetles (Coleoptera, Meloidae) and two large intergenic spacers in Hycleus genera
Source: BMC Genomics. 2017 Sep 6;18:698. doi: 10.1186/s12864-017-4102-y (PMC5585954; doi:10.1186/s12864-017-4102-y)
Supplement: Supplementary file 1 — Annotation of the Mylabris aulica mitogenome. (DOCX 21 kb) [file 12864_2017_4102_MOESM1_ESM.docx]

Additional file 1: Table S1. Annotation of the *Mylabris aulica* mitogenome

| Gene | Strand | Location | Size | Inc | Anticodon | Start codon | Stop codon |
| --- | --- | --- | --- | --- | --- | --- | --- |
| *trnI* | J | 1-66 | 66 |  | GAT |  |  |
| *trnQ* | N | 64-132 | 69 | -3 | TTG |  |  |
| *trnM* | J | 132-200 | 69 | -1 | CAT |  |  |
| *nad2* | J | 201-1214 | 1014 |  |  | ATA | TAA |
| *trnW* | J | 1213-1281 | 69 | -2 | TCA |  |  |
| *trnC* | N | 1284-1347 | 64 | 2 | GCA |  |  |
| *trnY* | N | 1350-1413 | 64 | 2 | GTA |  |  |
| *cox1* | J | 1406-2948 | 1543 | -8 |  | ATT | T(AA)* |
| *trnL(UUR)* | J | 2949-3013 | 65 |  | TAA |  |  |
| *cox2* | J | 3014-3701 | 688 |  |  | ATA | T(AA)* |
| *trnK* | J | 3702-3772 | 71 |  | CTT |  |  |
| *trnD* | J | 3772-3836 | 65 | -1 | GTC |  |  |
| *atp8* | J | 3837-3998 | 162 |  |  | ATT | TAA |
| *atp6* | J | 3989-4660 | 672 | -10 |  | ATG | TAA |
| *cox3* | J | 4660-5442 | 783 | -1 |  | ATG | TAG |
| *trnG* | J | 5444-5508 | 65 | 1 | TCC |  |  |
| *nad3* | J | 5506-5862 | 357 | -3 |  | ATA | TAA |
| *trnA* | J | 5866-5930 | 65 | 3 | TGC |  |  |
| *trnR* | J | 5930-5992 | 64 | -1 | TCG |  |  |
| *trnN* | J | 5993-6058 | 66 |  | GTT |  |  |
| *trnS(AGN)* | J | 6059-6116 | 58 |  | TCT |  |  |
| *trnE* | J | 6120-6181 | 62 | 3 | TTC |  |  |
| *trnF* | N | 6180-6243 | 64 | -2 | GAA |  |  |
| *nad5* | N | 6244-7954 | 1711 |  |  | ATT | T(AA)* |
| *trnH* | N | 7955-8016 | 62 |  | GTG |  |  |
| *nad4* | N | 8017-9349 | 1333 |  |  | ATG | T(AA)* |
| *nad4L* | N | 9343-9630 | 288 | -7 |  | ATG | TAA |
| *trnT* | J | 9633-9696 | 64 | 2 | TGT |  |  |
| *trnP* | N | 9697-9759 | 63 |  | TGG |  |  |
| *nad6* | J | 9762-10256 | 495 | 2 |  | ATT | TAA |
| *cob* | J | 10256-11393 | 1138 | -1 |  | ATG | TAA |
| *trnS(UCN)* | J | 11394-11461 | 68 |  | TGA |  |  |
| *nad1* | N | 11479-12429 | 951 | 17 |  | ATT | TAG |
| *trnL(CUN)* | N | 12430-12493 | 64 |  | TAG |  |  |
| *rrnL* | N | 12494-13767 | 1275 |  |  |  |  |
| *trnV* | N | 13768-13836 | 69 |  | TAC |  |  |
| *rrnS* | N | 13837-14619 | 791 |  |  |  |  |
| control region |  | 14620-15758 | 1139 |  |  |  |  |

**Inc**: intergenic nucleotides, negative values refer to overlapping nucleotides.

*TAA stop codon is completed by the addition of 3' A residues to the mRNA.
