# Supplementary material for: Mitochondrial genomes of blister beetles (Coleoptera, Meloidae) and two large intergenic spacers in Hycleus genera
Source: BMC Genomics. 2017 Sep 6;18:698. doi: 10.1186/s12864-017-4102-y (PMC5585954; doi:10.1186/s12864-017-4102-y)
Supplement: Supplementary file 3 — Annotation of the Hycleus marcipoli mitogenome. (DOCX 21 kb) [file 12864_2017_4102_MOESM3_ESM.docx]

Additional file 3: Table S3. Annotation of the *Hycleus marcipoli* mitogenome

| Gene | Strand | Location | Size | Inc | Anticodon | Start codon | Stop codon |
| --- | --- | --- | --- | --- | --- | --- | --- |
| *trnI* | J | 1-65 | 65 |  | GAT |  |  |
| *trnQ* | N | 63-131 | 69 | -3 | TTG |  |  |
| *trnM* | J | 131-199 | 69 | -1 | CAT |  |  |
| *nad2* | J | 200-1213 | 1014 |  |  | ATA | TAA |
| *trnW* | J | 1213-1280 | 68 | -1 | TCA |  |  |
| *trnC* | N | 1337-1400 | 64 | 56 | GCA |  |  |
| *trnY* | N | 1404-1469 | 66 | 3 | GTA |  |  |
| *cox1* | J | 1462-3004 | 1543 | -8 |  | ATT | T(AA)* |
| *trnL(UUR)* | J | 3005-3069 | 65 |  | TAA |  |  |
| *cox2* | J | 3070-3757 | 688 |  |  | ATA | T(AA)* |
| *trnK* | J | 3758-3828 | 71 | 4 | CTT |  |  |
| *trnD* | J | 3829-3893 | 65 |  | GTC |  |  |
| *atp8* | J | 3894-4055 | 162 |  |  | ATT | TAA |
| *atp6* | J | 4046-4717 | 672 | -10 |  | ATG | TAA |
| *cox3* | J | 4717-5499 | 783 | -1 |  | ATG | TAG |
| *trnG* | J | 5515-5578 | 64 | 15 | TCC |  |  |
| *nad3* | J | 5576-5932 | 357 | -3 |  | ATA | TAG |
| *trnA* | J | 5931-5995 | 65 | -2 | TGC |  |  |
| *trnR* | J | 5995-6060 | 66 | -1 | TCG |  |  |
| *trnN* | J | 6061-6126 | 66 |  | GTT |  |  |
| *trnS(AGN)* | J | 6127-6185 | 59 |  | TCT |  |  |
| *trnE* | J | 6188-6249 | 62 | 2 | TTC |  |  |
| *trnF* | N | 6248-6312 | 65 | -2 | GAA |  |  |
| *nad5* | N | 6313-8023 | 1711 |  |  | ATT | T(AA)* |
| *trnH* | N | 8024-8088 | 65 |  | GTG |  |  |
| *nad4* | N | 8089-9421 | 1333 |  |  | ATG | T(AA)* |
| *nad4L* | N | 9415-9702 | 288 | -7 |  | ATG | TAA |
| *trnT* | J | 9705-9767 | 63 | 2 | TGT |  |  |
| *trnP* | N | 9768-9831 | 64 |  | TGG |  |  |
| *nad6* | J | 9834-10325 | 492 | 2 |  | ATT | TAA |
| *cob* | J | 10325-11464 | 1140 | -1 |  | ATG | TAA |
| *trnS(UCN)* | J | 11463-11530 | 68 | -2 | TGA |  |  |
| *nad1* | N | 11653-12603 | 951 | 122 |  | ATT | TAG |
| *trnL(CUN)* | N | 12604-12667 | 64 |  | TAG |  |  |
| *rrnL* | N | 12668-13943 | 1276 |  |  |  |  |
| *trnV* | N | 13944-14012 | 69 |  | TAC |  |  |
| *rrnS* | N | 14013-14798 | 786 |  |  |  |  |
| control region |  | 14799-15923 | 1125 |  |  |  |  |

**Inc**: intergenic nucleotides, negative values refer to overlapping nucleotides.

*TAA stop codon is completed by the addition of 3' A residues to the mRNA.
