# Supplementary material for: Mitochondrial genomes of blister beetles (Coleoptera, Meloidae) and two large intergenic spacers in Hycleus genera
Source: BMC Genomics. 2017 Sep 6;18:698. doi: 10.1186/s12864-017-4102-y (PMC5585954; doi:10.1186/s12864-017-4102-y)
Supplement: Supplementary file 4 — Annotation of the Hycleus cichorii mitogenome. (DOCX 22 kb) [file 12864_2017_4102_MOESM4_ESM.docx]

Additional file 4: Table S4. Annotation of the *Hycleus cichorii* mitogenome

| Gene | Strand | Location | Size | Inc | Anticodon | Start codon | Stop codon |
| --- | --- | --- | --- | --- | --- | --- | --- |
| *trnI* | J |  |  |  | GAT |  |  |
| *trnQ* | N |  |  |  | TTG |  |  |
| *trnM* | J | 1-17 |  |  | CAT |  |  |
| *nad2* | J | 18-1031 | 1014 |  |  | ATA | TAA |
| *trnW* | J | 1034-1099 | 66 | 2 | TCA |  |  |
| *trnC* | N | 1139-1202 | 64 | 39 | GCA |  |  |
| *trnY* | N | 1206-1271 | 66 | 3 | GTA |  |  |
| *cox1* | J | 1264-2806 | 1543 | -8 |  | ATT | T(AA)* |
| *trnL(UUR)* | J | 2807-2871 | 65 |  | TAA |  |  |
| *cox2* | J | 2872-3559 | 688 |  |  | ATA | T(AA)* |
| *trnK* | J | 3560-3630 | 71 |  | CTT |  |  |
| *trnD* | J | 3631-3695 | 66 |  | GTC |  |  |
| *atp8* | J | 3696-3857 | 162 |  |  | ATT | TAA |
| *atp6* | J | 3848-4519 | 672 | -10 |  | ATG | TAA |
| *cox3* | J | 4519-5301 | 783 | -1 |  | ATG | TAG |
| *trnG* | J | 5317-5380 | 64 | 15 | TCC |  |  |
| *nad3* | J | 5378-5734 | 357 | -3 |  | ATA | TAA |
| *trnA* | J | 5733-5797 | 65 | -2 | TGC |  |  |
| *trnR* | J | 5797-5863 | 67 | -1 | TCG |  |  |
| *trnN* | J | 5864-5929 | 65 |  | GTT |  |  |
| *trnS(AGN)* | J | 5930-5988 | 59 |  | TCT |  |  |
| *trnE* | J | 5991-6052 | 62 | 2 | TTC |  |  |
| *trnF* | N | 6051-6115 | 65 | -2 | GAA |  |  |
| *nad5* | N | 6116-7826 | 1711 |  |  | ATT | T(AA)* |
| *trnH* | N | 7827-7891 | 65 |  | GTG |  |  |
| *nad4* | N | 7892-9224 | 1333 |  |  | ATG | T(AA)* |
| *nad4L* | N | 9218-9505 | 288 | -7 |  | ATG | TAA |
| *trnT* | J | 9508-9570 | 63 | 2 | TGT |  |  |
| *trnP* | N | 9571-9633 | 64 |  | TGG |  |  |
| *nad6* | J | 9636-10127 | 492 | 2 |  | ATT | TAA |
| *cob* | J | 10127-11266 | 1140 | -1 |  | ATG | TAA |
| *trnS(UCN)* | J | 11265-11332 | 68 | -2 | TGA |  |  |
| *nad1* | N | 11443-12393 | 951 | 181 |  | ATT | TAG |
| *trnL(CUN)* | N | 12394-12457 | 65 |  | TAG |  |  |
| *rrnL* | N | 12458-13735 | 1281 |  |  |  |  |
| *trnV* | N | 13736-13804 | 69 |  | TAC |  |  |
| *rrnS* | N | 13805-14370 | 566 |  |  |  |  |
| control region |  |  |  |  |  |  |  |

**Inc**: intergenic nucleotides, negative values refer to overlapping nucleotides.

*TAA stop codon is completed by the addition of 3' A residues to the mRNA.
