# Supplementary material for: Mitochondrial genomes of blister beetles (Coleoptera, Meloidae) and two large intergenic spacers in Hycleus genera
Source: BMC Genomics. 2017 Sep 6;18:698. doi: 10.1186/s12864-017-4102-y (PMC5585954; doi:10.1186/s12864-017-4102-y)
Supplement: Supplementary file 5 — Annotation of the Epicauta gorhami mitogenome. (DOCX 21 kb) [file 12864_2017_4102_MOESM5_ESM.docx]

Additional file 5: Table S5. Annotation of the *Epicauta gorhami* mitogenome

| Gene | Strand | Location | Size | Inc | Anticodon | Start codon | Stop codon |
| --- | --- | --- | --- | --- | --- | --- | --- |
| *trnI* | J | 1-66 | 66 |  | GAT |  |  |
| *trnQ* | N | 64-132 | 69 | -3 | TTG |  |  |
| *trnM* | J | 132-200 | 69 | -1 | CAT |  |  |
| *nad2* | J | 201-1214 | 1014 |  |  | ATA | TAA |
| *trnW* | J | 1213-1280 | 68 | -2 | TCA |  |  |
| *trnC* | N | 1280-1343 | 64 | -1 | GCA |  |  |
| *trnY* | N | 1346-1409 | 66 | 2 | GTA |  |  |
| *cox1* | J | 1402-2944 | 1543 | -9 |  | ATT | T(AA)* |
| *trnL(UUR)* | J | 2945-3009 | 65 |  | TAA |  |  |
| *cox2* | J | 3010-3697 | 688 |  |  | ATA | T(AA)* |
| *trnK* | J | 3698-3768 | 71 |  | CTT |  |  |
| *trnD* | J | 3768-3823 | 65 | -1 | GTC |  |  |
| *atp8* | J | 3833-3994 | 162 | 9 |  | ATT | TAA |
| *atp6* | J | 3985-4656 | 672 | -10 |  | ATG | TAA |
| *cox3* | J | 4656-5436 | 781 | -1 |  | ATG | T(AA)* |
| *trnG* | J | 5437-5500 | 64 |  | TCC |  |  |
| *nad3* | J | 5498-5854 | 357 | -3 |  | ATA | TAG |
| *trnA* | J | 5853-5917 | 65 | -2 | TGC |  |  |
| *trnR* | J | 5917-5980 | 64 | -1 | TCG |  |  |
| *trnN* | J | 5980-6044 | 65 | -1 | GTT |  |  |
| *trnS(AGN)* | J | 6045-6101 | 57 |  | TCT |  |  |
| *trnE* | J | 6102-6163 | 62 | 2 | TTC |  |  |
| *trnF* | N | 6162-6224 | 63 | -2 | GAA |  |  |
| *nad5* | N | 6225-7935 | 1711 |  |  | ATT | T(AA)* |
| *trnH* | N | 7936-7999 | 64 |  | GTG |  |  |
| *nad4* | N | 8000-9332 | 1333 |  |  | ATG | T(AA)* |
| *nad4L* | N | 9326-9613 | 288 | -7 |  | ATG | TAA |
| *trnT* | J | 9616-9678 | 63 | 2 | TGT |  |  |
| *trnP* | N | 9679-9742 | 64 |  | TGG |  |  |
| *nad6* | J | 9745-10236 | 492 | 2 |  | ATT | TAA |
| *cob* | J | 10236-11375 | 1140 | -1 |  | ATG | TAA |
| *trnS(UCN)* | J | 11374-11441 | 68 | -2 | TGA |  |  |
| *nad1* | N | 11459-12409 | 951 | 17 |  | ATT | TAG |
| *trnL(CUN)* | N | 12410-12473 | 64 |  | TAG |  |  |
| *rrnL* | N | 12474-13750 | 1277 |  |  |  |  |
| *trnV* | N | 13751-13819 | 63 |  | TAC |  |  |
| *rrnS* | N | 13820-14608 | 787 |  |  |  |  |
| control region |  | 14609-15691 | 1083 |  |  |  |  |

**Inc**: intergenic nucleotides, negative values refer to overlapping nucleotides.

*TAA stop codon is completed by the addition of 3' A residues to the mRNA.
