# Supplementary material for: Mitochondrial genomes of blister beetles (Coleoptera, Meloidae) and two large intergenic spacers in Hycleus genera
Source: BMC Genomics. 2017 Sep 6;18:698. doi: 10.1186/s12864-017-4102-y (PMC5585954; doi:10.1186/s12864-017-4102-y)
Supplement: Supplementary file 6 — Annotation of the Epicauta tibialis mitogenome. (DOCX 21 kb) [file 12864_2017_4102_MOESM6_ESM.docx]

Additional file 6: Table S6. Annotation of the *Epicauta tibialis* mitogenome

| Gene | Strand | Location | Size | Inc | Anticodon | Start codon | Stop codon |
| --- | --- | --- | --- | --- | --- | --- | --- |
| *trnI* | J | 1-66 | 66 |  | GAT |  |  |
| *trnQ* | N | 64-132 | 69 | -3 | TTG |  |  |
| *trnM* | J | 132-200 | 69 | -1 | CAT |  |  |
| *nad2* | J | 201-1214 | 1014 |  |  | ATA | TAA |
| *trnW* | J | 1213-1280 | 68 | -2 | TCA |  |  |
| *trnC* | N | 1280-1343 | 64 | -1 | GCA |  |  |
| *trnY* | N | 1349-1412 | 64 | 5 | GTA |  |  |
| *cox1* | J | 1405-2947 | 1543 | -8 |  | ATT | T(AA)* |
| *trnL(UUR)* | J | 2948-3012 | 65 |  | TAA |  |  |
| *cox2* | J | 3013-3700 | 688 |  |  | ATA | T(AA)* |
| *trnK* | J | 3701-3771 | 71 |  | CTT |  |  |
| *trnD* | J | 3771-3836 | 66 | -1 | GTC |  |  |
| *atp8* | J | 3837-3998 | 162 |  |  | ATT | TAA |
| *atp6* | J | 3989-4660 | 672 | -10 |  | ATG | TAA |
| *cox3* | J | 4660-5440 | 781 | -1 |  | ATG | T(AA)* |
| *trnG* | J | 5441-5504 | 64 |  | TCC |  |  |
| *nad3* | J | 5502-5858 | 357 | -3 |  | ATA | TAG |
| *trnA* | J | 5857-5921 | 65 | -2 | TGC |  |  |
| *trnR* | J | 5921-5983 | 63 | -1 | TCG |  |  |
| *trnN* | J | 5984-6048 | 65 | 9 | GTT |  |  |
| *trnS(AGN)* | J | 6049-6105 | 58 |  | TCT |  |  |
| *trnE* | J | 6106-6167 | 62 | 2 | TTC |  |  |
| *trnF* | N | 6166-6228 | 66 | -2 | GAA |  |  |
| *nad5* | N | 6229-7939 | 1711 |  |  | ATT | T(AA)* |
| *trnH* | N | 7940-8003 | 65 |  | GTG |  |  |
| *nad4* | N | 8004-9336 | 1333 |  |  | ATG | T(AA)* |
| *nad4L* | N | 9330-9617 | 288 | -5 |  | ATG | TAA |
| *trnT* | J | 9620-9683 | 64 | 2 | TGT |  |  |
| *trnP* | N | 9684-9747 | 64 |  | TGG |  |  |
| *nad6* | J | 9750-10241 | 492 | 2 |  | ATT | TAA |
| *cob* | J | 10241-11380 | 1140 | -1 |  | ATG | TAA |
| *trnS(UCN)* | J | 11379-11446 | 68 | -2 | TGA |  |  |
| *nad1* | N | 11464-12414 | 951 | 17 |  | ATT | TAG |
| *trnL(CUN)* | N | 12415-12478 | 63 |  | TAG |  |  |
| *rrnL* | N | 12479-13757 | 1279 |  |  |  |  |
| *trnV* | N | 13758-13826 | 69 |  | TAC |  |  |
| *rrnS* | N | 13827-14617 | 792 |  |  |  |  |
| control region |  | 14618-15716 | 1099 |  |  |  |  |

**Inc**: intergenic nucleotides, negative values refer to overlapping nucleotides.

*TAA stop codon is completed by the addition of 3' A residues to the mRNA.
