# Supplementary material for: Mitochondrial genomes of blister beetles (Coleoptera, Meloidae) and two large intergenic spacers in Hycleus genera
Source: BMC Genomics. 2017 Sep 6;18:698. doi: 10.1186/s12864-017-4102-y (PMC5585954; doi:10.1186/s12864-017-4102-y)
Supplement: Supplementary file 8 — Codon usage in mitochondrial genomes of seven meloids. (DOCX 27 kb) [file 12864_2017_4102_MOESM8_ESM.docx]

Additional file 8: Table S8. Codon usage in mitochondrial genomes of seven meloids

| Codon(AA) | *H.phaleratus* | | *H.marcipoli* | | *H.cichorii* | | *M. aulica* | | *E.gorhami* | | *E.tibialis* | | *L.caraganae* | |
| --- | --- | --- | --- | --- | --- | --- | --- | --- | --- | --- | --- | --- | --- | --- |
|  | Count | RSCU | Count | RSCU | Count | RSCU | Count | RSCU | Count | RSCU | Count | RSCU | Count | RSCU |
| UUU(F) | 205 | 1.29 | 224 | 1.42 | 222 | 1.37 | 214 | 1.35 | 203 | 1.31 | 216 | 1.37 | 229 | 1.45 |
| UUC(F) | 112 | 0.71 | 92 | 0.58 | 101 | 0.63 | 103 | 0.65 | 106 | 0.69 | 99 | 0.63 | 86 | 0.55 |
| UUA(L) | 225 | 2.28 | 284 | 2.91 | 239 | 2.44 | 228 | 2.3 | 248 | 2.51 | 205 | 2.1 | 278 | 2.87 |
| UUG(L) | 86 | 0.87 | 58 | 0.59 | 80 | 0.82 | 50 | 0.5 | 69 | 0.7 | 92 | 0.94 | 46 | 0.48 |
| CUU(L) | 97 | 0.98 | 104 | 1.06 | 93 | 0.95 | 125 | 1.26 | 111 | 1.12 | 107 | 1.09 | 108 | 1.12 |
| CUC(L) | 40 | 0.4 | 27 | 0.28 | 35 | 0.36 | 41 | 0.41 | 21 | 0.21 | 37 | 0.38 | 23 | 0.24 |
| CUA(L) | 120 | 1.21 | 97 | 0.99 | 108 | 1.1 | 112 | 1.13 | 117 | 1.18 | 106 | 1.08 | 102 | 1.05 |
| CUG(L) | 25 | 0.25 | 16 | 0.16 | 33 | 0.34 | 40 | 0.4 | 27 | 0.27 | 40 | 0.41 | 24 | 0.25 |
| AUU(I) | 258 | 1.36 | 294 | 1.49 | 277 | 1.46 | 252 | 1.45 | 262 | 1.46 | 235 | 1.37 | 286 | 1.52 |
| AUC(I) | 121 | 0.64 | 100 | 0.51 | 102 | 0.54 | 96 | 0.55 | 97 | 0.54 | 107 | 0.63 | 91 | 0.48 |
| AUA(M) | 178 | 1.45 | 204 | 1.59 | 172 | 1.39 | 175 | 1.44 | 201 | 1.49 | 186 | 1.48 | 217 | 1.67 |
| AUG(M) | 67 | 0.55 | 53 | 0.41 | 75 | 0.61 | 68 | 0.56 | 69 | 0.51 | 65 | 0.52 | 43 | 0.33 |
| GUU(V) | 90 | 1.78 | 83 | 1.75 | 73 | 1.47 | 82 | 1.43 | 72 | 1.42 | 97 | 1.65 | 70 | 1.37 |
| GUC(V) | 21 | 0.42 | 15 | 0.32 | 29 | 0.58 | 27 | 0.47 | 24 | 0.47 | 31 | 0.53 | 18 | 0.35 |
| GUA(V) | 66 | 1.31 | 78 | 1.64 | 71 | 1.43 | 93 | 1.62 | 74 | 1.46 | 81 | 1.38 | 96 | 1.87 |
| GUG(V) | 25 | 0.5 | 14 | 0.29 | 26 | 0.52 | 27 | 0.47 | 33 | 0.65 | 26 | 0.44 | 21 | 0.41 |
| UAU(Y) | 122 | 1.52 | 119 | 1.5 | 109 | 1.36 | 108 | 1.42 | 93 | 1.22 | 90 | 1.23 | 106 | 1.34 |
| UAC(Y) | 38 | 0.47 | 40 | 0.5 | 51 | 0.64 | 44 | 0.58 | 59 | 0.78 | 56 | 0.77 | 52 | 0.66 |
| CAU(H) | 59 | 1.64 | 59 | 1.69 | 60 | 1.67 | 51 | 1.36 | 48 | 1.25 | 53 | 1.32 | 54 | 1.5 |
| CAC(H) | 13 | 0.36 | 11 | 0.31 | 12 | 0.33 | 24 | 0.64 | 29 | 0.75 | 27 | 0.68 | 18 | 0.5 |
| CAA(Q) | 53 | 1.43 | 58 | 1.59 | 56 | 1.51 | 49 | 1.4 | 37 | 1.19 | 36 | 1.11 | 54 | 1.59 |
| CAG(Q) | 21 | 0.57 | 15 | 0.41 | 18 | 0.49 | 21 | 0.6 | 25 | 0.81 | 29 | 0.89 | 14 | 0.41 |
| AAU(N) | 119 | 1.32 | 138 | 1.5 | 109 | 1.25 | 125 | 1.38 | 114 | 1.3 | 109 | 1.25 | 132 | 1.43 |
| AAC(N) | 61 | 0.68 | 46 | 0.5 | 66 | 0.75 | 56 | 0.62 | 62 | 0.7 | 65 | 0.75 | 53 | 0.57 |
| AAA(K) | 72 | 1.33 | 75 | 1.4 | 65 | 1.24 | 45 | 0.97 | 59 | 1.13 | 71 | 1.38 | 66 | 1.27 |
| AAG(K) | 36 | 0.67 | 32 | 0.6 | 40 | 0.76 | 48 | 1.03 | 45 | 0.87 | 32 | 0.62 | 38 | 0.73 |
| GAU(D) | 49 | 1.32 | 53 | 1.56 | 51 | 1.42 | 41 | 1.22 | 43 | 1.16 | 35 | 1 | 39 | 1.16 |
| GAC(D) | 25 | 0.68 | 15 | 0.44 | 21 | 0.58 | 26 | 0.78 | 31 | 0.84 | 35 | 1 | 28 | 0.84 |
| GAA(E) | 60 | 1.48 | 69 | 1.62 | 64 | 1.56 | 51 | 1.38 | 54 | 1.44 | 57 | 1.44 | 57 | 1.43 |
| GAG(E) | 21 | 0.52 | 16 | 0.38 | 18 | 0.44 | 23 | 0.62 | 21 | 0.56 | 22 | 0.56 | 23 | 0.57 |
| UCU(S) | 90 | 2.06 | 87 | 2.04 | 92 | 2.11 | 112 | 2.55 | 101 | 2.33 | 93 | 2.18 | 100 | 2.23 |
| UCC(S) | 42 | 0.96 | 37 | 0.87 | 48 | 1.1 | 32 | 0.73 | 32 | 0.74 | 41 | 0.96 | 49 | 1.09 |
| UCA(S) | 96 | 2.19 | 101 | 2.37 | 88 | 2.02 | 75 | 1.71 | 79 | 1.82 | 63 | 1.48 | 78 | 1.74 |
| UCG(S) | 9 | 0.21 | 5 | 0.12 | 9 | 0.21 | 12 | 0.27 | 16 | 0.37 | 25 | 0.59 | 5 | 0.11 |
| CCU(P) | 71 | 2.1 | 74 | 2.18 | 69 | 2.06 | 79 | 2.27 | 67 | 2 | 46 | 1.34 | 67 | 1.99 |
| CCC(P) | 21 | 0.62 | 22 | 0.65 | 25 | 0.75 | 26 | 0.75 | 23 | 0.69 | 39 | 1.14 | 24 | 0.71 |
| CCA(P) | 41 | 1.21 | 37 | 1.09 | 35 | 1.04 | 30 | 0.86 | 31 | 0.93 | 38 | 1.11 | 37 | 1.1 |
| CCG(P) | 2 | 0.06 | 3 | 0.09 | 5 | 0.15 | 4 | 0.12 | 13 | 0.39 | 14 | 0.41 | 7 | 0.21 |
| ACU(T) | 60 | 1.48 | 74 | 1.79 | 69 | 1.61 | 71 | 1.54 | 83 | 1.83 | 80 | 1.66 | 73 | 1.63 |
| ACC(T) | 39 | 0.96 | 34 | 0.82 | 35 | 0.82 | 37 | 0.8 | 34 | 0.75 | 49 | 1.02 | 47 | 1.05 |
| ACA(T) | 59 | 1.46 | 52 | 1.26 | 60 | 1.4 | 70 | 1.52 | 57 | 1.26 | 56 | 1.16 | 52 | 1.16 |
| ACG(T) | 4 | 0.1 | 5 | 0.12 | 7 | 0.16 | 6 | 0.13 | 7 | 0.15 | 8 | 0.17 | 7 | 0.16 |
| GCU(A) | 66 | 1.54 | 75 | 1.74 | 77 | 1.79 | 76 | 1.66 | 85 | 1.87 | 60 | 1.35 | 69 | 1.68 |
| GCC(A) | 48 | 1.12 | 34 | 0.79 | 42 | 0.98 | 45 | 0.98 | 41 | 0.9 | 57 | 1.28 | 42 | 1.02 |
| GCA(A) | 48 | 1.12 | 56 | 1.3 | 42 | 0.98 | 54 | 1.18 | 44 | 0.97 | 39 | 0.88 | 46 | 1.12 |
| GCG(A) | 9 | 0.21 | 7 | 0.16 | 11 | 0.26 | 8 | 0.17 | 12 | 0.26 | 22 | 0.49 | 7 | 0.17 |
| UGU(C) | 27 | 1.46 | 31 | 1.63 | 28 | 1.44 | 22 | 1.38 | 27 | 1.46 | 26 | 1.58 | 25 | 1.52 |
| UGC(C) | 10 | 0.54 | 7 | 0.37 | 11 | 0.56 | 10 | 0.63 | 10 | 0.54 | 7 | 0.42 | 8 | 0.48 |
| UGA(W) | 72 | 1.5 | 83 | 1.73 | 64 | 1.36 | 67 | 1.44 | 66 | 1.4 | 69 | 1.42 | 66 | 1.4 |
| UGG(W) | 24 | 0.5 | 13 | 0.27 | 30 | 0.64 | 26 | 0.56 | 28 | 0.6 | 28 | 0.58 | 28 | 0.6 |
| CGU(R) | 19 | 1.31 | 21 | 1.42 | 21 | 1.42 | 19 | 1.29 | 21 | 1.47 | 19 | 1.36 | 21 | 1.53 |
| CGC(R) | 8 | 0.55 | 5 | 0.34 | 9 | 0.61 | 6 | 0.41 | 6 | 0.42 | 7 | 0.5 | 4 | 0.29 |
| CGA(R) | 16 | 1.1 | 26 | 1.76 | 20 | 1.36 | 26 | 1.76 | 18 | 1.26 | 19 | 1.36 | 19 | 1.38 |
| CGG(R) | 15 | 1.03 | 7 | 0.47 | 9 | 0.61 | 8 | 0.54 | 12 | 0.84 | 11 | 0.79 | 11 | 0.8 |
| AGU(S) | 26 | 0.59 | 26 | 0.61 | 26 | 0.6 | 31 | 0.71 | 34 | 0.78 | 30 | 0.7 | 30 | 0.67 |
| AGC(S) | 11 | 0.25 | 7 | 0.16 | 8 | 0.18 | 18 | 0.41 | 20 | 0.46 | 19 | 0.45 | 18 | 0.4 |
| AGA(S) | 67 | 1.53 | 64 | 1.5 | 66 | 1.51 | 57 | 1.3 | 52 | 1.2 | 46 | 1.08 | 55 | 1.23 |
| AGG(S) | 9 | 0.21 | 14 | 0.33 | 12 | 0.28 | 14 | 0.32 | 13 | 0.3 | 24 | 0.56 | 23 | 0.51 |
| GGU(G) | 68 | 1.31 | 81 | 1.6 | 54 | 1.04 | 50 | 0.93 | 44 | 0.82 | 32 | 0.58 | 46 | 0.88 |
| GGC(G) | 17 | 0.33 | 7 | 0.14 | 20 | 0.39 | 29 | 0.54 | 31 | 0.58 | 37 | 0.68 | 18 | 0.34 |
| GGA(G) | 78 | 1.51 | 78 | 1.54 | 83 | 1.6 | 77 | 1.43 | 60 | 1.12 | 61 | 1.11 | 87 | 1.66 |
| GGG(G) | 44 | 0.85 | 37 | 0.73 | 50 | 0.97 | 60 | 1.11 | 80 | 1.49 | 89 | 1.63 | 59 | 1.12 |

Average 3,699 codons for meloids were analyzed, excluding the stop codons. AA, amino acid; RSCU, relative synonymous codon usage.
