# Supplementary material for: Mitochondrial genomes of blister beetles (Coleoptera, Meloidae) and two large intergenic spacers in Hycleus genera
Source: BMC Genomics. 2017 Sep 6;18:698. doi: 10.1186/s12864-017-4102-y (PMC5585954; doi:10.1186/s12864-017-4102-y)
Supplement: Supplementary file 9 — Summary of multiple alignments of tRNAs in meloid mitogenomes. (DOCX 15 kb) [file 12864_2017_4102_MOESM9_ESM.docx]

Additional file 9: Table S9. Summary of multiple alignments of tRNAs in meloid mitogenomes.

| Alignment name | Amino acid | Alignment length | Identical positions | Percent of identical nucleotides (%) |
| --- | --- | --- | --- | --- |
| *trnA* | Alanine | 66 | 59 | 89.39 |
| *trnE* | Glutamate | 62 | 54 | 87.10 |
| *trnG* | Glycine | 64 | 54 | 84.38 |
| *trnK* | Lysine | 72 | 59 | 81.94 |
| *trnY* | Tyrosine | 65 | 53 | 81.54 |
| *trnM** | Methionine | 69 | 56 | 81.16 |
| *trnL2* | Leucine (UUR) | 65 | 52 | 80.00 |
| *trnS2* | Serine (UCN) | 68 | 54 | 79.41 |
| *trnC* | Cysteine | 64 | 50 | 78.13 |
| *trnN* | Asparagine | 68 | 53 | 77.94 |
| *trnV* | Valine | 70 | 52 | 74.29 |
| *trnW* | Tryptophan | 68 | 50 | 73.53 |
| *trnS1* | Serine (AGN) | 59 | 43 | 72.88 |
| *trnP* | Proline | 65 | 47 | 72.31 |
| *trnD* | Aspartate | 66 | 47 | 71.21 |
| *trnT* | Threonine | 64 | 45 | 70.31 |
| *trnL1* | Leucine (CUN) | 66 | 44 | 66.67 |
| *trnI** | Isoleucine | 66 | 38 | 57.58 |
| *trnR* | Arginine | 68 | 37 | 54.41 |
| *trnF* | Phenylalanine | 66 | 35 | 53.03 |
| *trnH* | Histidine | 68 | 36 | 52.94 |
| *trnQ** | Glutamine | 69 | 34 | 49.28 |

*absent in mitogenome of *H. cichorii*.
