# Supplementary figures and images for: Mitochondrial genomes of blister beetles (Coleoptera, Meloidae) and two large intergenic spacers in Hycleus genera
Source: BMC Genomics. 2017 Sep 6;18:698. doi: 10.1186/s12864-017-4102-y (PMC5585954; doi:10.1186/s12864-017-4102-y)

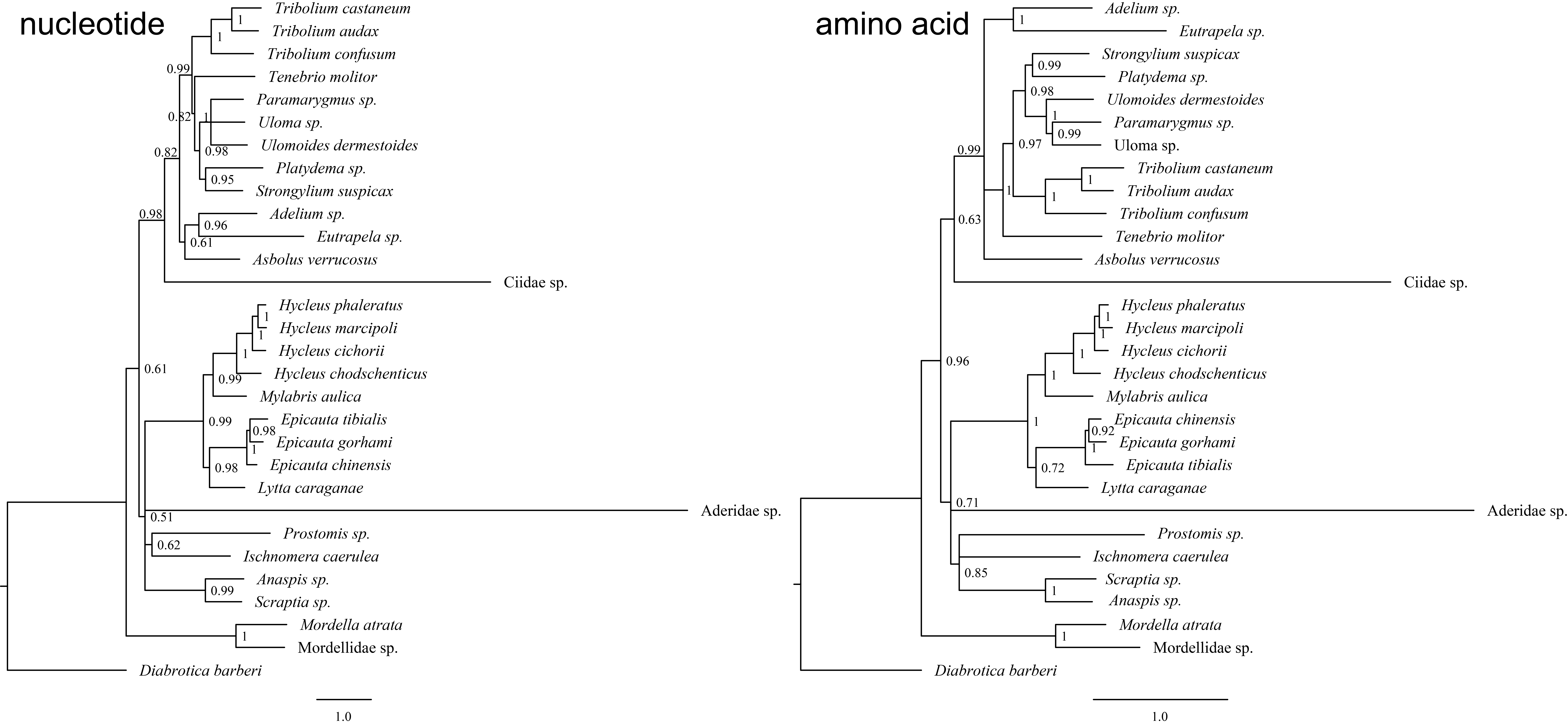

Supplement: Supplementary file 10 — The phylogenetic tree of 16 species from superfamily Tenebrionoidea based on the nucleotide dataset and the amino acids dataset of 13 mitochondrial protein-coding genes, inferred from PhyloBayes. The numbers abutting branches refer to Bayesian posterior probabilities. The Diabrotica barberi (Coleoptera: Chrysomelidae) was employed to root the trees as outgroup. (TIFF 1285 kb) [file 12864_2017_4102_MOESM10_ESM.tif]
