# Supplementary material for: Mitochondrial genomes of blister beetles (Coleoptera, Meloidae) and two large intergenic spacers in Hycleus genera
Source: BMC Genomics. 2017 Sep 6;18:698. doi: 10.1186/s12864-017-4102-y (PMC5585954; doi:10.1186/s12864-017-4102-y)
Supplement: Supplementary file 11 — PCR primers used to amplify the mitochondrial genomes of seven meloids. (DOCX 19 kb) [file 12864_2017_4102_MOESM11_ESM.docx]

Additional file 11: Table S10. PCR primers used to amplify the mitochondrial genomes of seven meloids

| Location | Length (kb) | Species | Primer pair | Primer sequence (5’-3’) |
| --- | --- | --- | --- | --- |
| *trnM-trnL* | 2.8 | All | C1F | AAGCTATTAGGTTCATACC |
|  |  |  | C1R | GCACTATTCTGCCATATTAGA |
| *cox1-cox2* | 1.5 | All | C2F | CAACATCTATTCTGATTCTTTGG |
|  |  |  | C2R | CAAATTTCTGAACATTG |
| *cox2-nad3* | 2.3 | All | C2N3F | GACTTAAACCCCAAATATAAAGG |
|  |  |  | C2N3R | ACGCTATTACTTAAGTTAGAAGCTTA |
| *nad3-nad4* | 3.1 | All | N34F | ATTATATTTGACTTCCAATCAAAAGA |
|  |  |  | N34R | ATTTGCTTACGCCAAAGAGAC |
| *nad4-nad1* | 3.4 | All | N41F | CCAGATGAACATAACCCATG |
|  |  |  | N41R | ATCGTACTCCATTTGATTTTGC |
| *nad1-rrnL* | 1.5 | All | N1LF | TTATCATAACGAAAACGAGG |
|  |  |  | N1LR | AGACGAGAAGACCCTATAGAG |
| *rrnL-**rrnS* | 2.2 | All | LSF | ATTGCATCTCTAAAAGGCTG |
|  |  |  | LSR | TGCCAGCAGTTGCGGTTATAC |
| *rrnS -cox1* | 1.8 | *H. phaleratus* | HPCRF | CCTCTGAATAGACTAAAATACCGCC |
|  |  |  | HPCRR | AGCAGCAGAATTAGGGAGGC |
|  |  | *H. marcipoli* | HMCRF | CCTCTGAATAGACTAAAATACCGCC |
|  |  |  | HMCRR | GATGAGGGAAAGCAGCAGAAT |
|  |  | *M. aulica* | MACRF | CCTCTGAATAGACTAAAATACCGCC |
|  |  |  | MACRR | CAGGAAATCAGTAGTGGAATGGG |
|  |  | *E. tibialis* | ETCRF | CTCTGAATAGACTAAAATACCGCCAA |
|  |  |  | ETCRR | TCAGGGAATCAGAAATGGAAAG |
|  |  | *E. gorhami* | EGCRF | TTCCTCTGAATAGATTAGAATACCGC |
|  |  |  | EGCRR | AAGAAAATGAGGAGATGGCGATA |
|  |  | *L. caraganae* | LCCRF | TTCTAATAATAGGGTATCTAATCCTAGTTT |
|  |  |  | LCCRR | TTAATCCTTCTATAACTTCAGGGAATCA |
