# Supplementary material for: Mitochondrial genomes of blister beetles (Coleoptera, Meloidae) and two large intergenic spacers in Hycleus genera
Source: BMC Genomics. 2017 Sep 6;18:698. doi: 10.1186/s12864-017-4102-y (PMC5585954; doi:10.1186/s12864-017-4102-y)
Supplement: Supplementary file 12 — The best-fit schemes and evolutionary models for two datasets. (DOCX 16 kb) [file 12864_2017_4102_MOESM12_ESM.docx]

Additional file 12: Table S11. The best-fit schemes and evolutionary models for two datasets.

| Data matrix | Subset | Best-fit scheme | Models |
| --- | --- | --- | --- |
| nucleotide | P1 | *atp8, nad2, nad6* | GTR+I+G |
|  | P2 | *atp6, cox1, cox2, cox3, cob, nad3* | GTR+I+G |
|  | P3 | *nad1, nad4, nad4L,nad5* | GTR+I+G |
| Amono acid | P1 | *atp8, nad2, nad3, nad6* | MtREV+I+G+F |
|  | P2 | *atp6, cox1,cox2, cox3,cob* | MtArt+I+G |
|  | P3 | *nad1, nad4, nad4L,nad5* | MtArt+I+G+F |
